# The Alzheimer’s disease-linked protease BACE1 modulates neuronal IL-6 signaling through shedding of the receptor gp130

**DOI:** 10.1186/s13024-023-00596-6

**Published:** 2023-02-21

**Authors:** Stephan A. Müller, Merav D. Shmueli, Xiao Feng, Johanna Tüshaus, Neele Schumacher, Ryan Clark, Brad E. Smith, An Chi, Stefan Rose-John, Matthew E. Kennedy, Stefan F. Lichtenthaler

**Affiliations:** 1 German Center for Neurodegenerative Diseases (DZNE), Munich, Germany; 2 Neuroproteomics, School of Medicine, Klinikum rechts der Isar, Technical University of Munich, Munich, Germany; 3 Biochemical Institute, Kiel University, Kiel, Germany; 4 Neuroscience, Merck & Co. Inc., Boston, MA USA; 5 Laboratory Animal Resources, Merck & Co. Inc., West Point, PA USA; 6 Chemical Biology, Merck & Co. Inc., Boston, MA USA; 7 Munich Cluster for Systems Neurology (SyNergy), Munich, Germany

**Keywords:** IL-6 receptor subunit beta, Secretase, IL-6R, Shedding, Trans-signaling, VCAM1

## Abstract

**Background:**

The protease BACE1 is a major drug target for Alzheimer’s disease, but chronic BACE1 inhibition is associated with non-progressive cognitive worsening that may be caused by modulation of unknown physiological BACE1 substrates.

**Methods:**

To identify in vivo-relevant BACE1 substrates, we applied pharmacoproteomics to non-human-primate cerebrospinal fluid (CSF) after acute treatment with BACE inhibitors.

**Results:**

Besides SEZ6, the strongest, dose-dependent reduction was observed for the pro-inflammatory cytokine receptor gp130/IL6ST, which we establish as an in vivo BACE1 substrate. Gp130 was also reduced in human CSF from a clinical trial with a BACE inhibitor and in plasma of BACE1-deficient mice. Mechanistically, we demonstrate that BACE1 directly cleaves gp130, thereby attenuating membrane-bound gp130 and increasing soluble gp130 abundance and controlling gp130 function in neuronal IL-6 signaling and neuronal survival upon growth-factor withdrawal.

**Conclusion:**

BACE1 is a new modulator of gp130 function. The BACE1-cleaved, soluble gp130 may serve as a pharmacodynamic BACE1 activity marker to reduce the occurrence of side effects of chronic BACE1 inhibition in humans.

**Graphical abstract:**

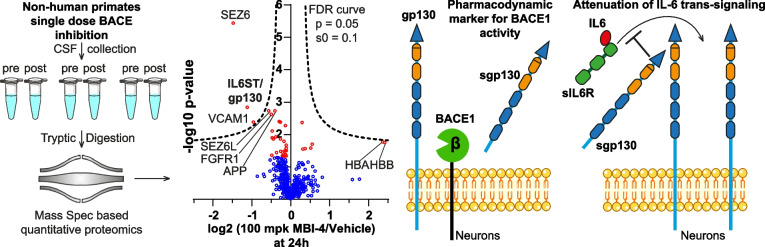

**Supplementary Information:**

The online version contains supplementary material available at 10.1186/s13024-023-00596-6.

## Background

The β-secretase BACE1 (β-site APP cleaving enzyme) is a major drug target in Alzheimer’s disease (AD) [1]. BACE1 proteolytically cleaves the amyloid precursor protein (APP) [2–5], thereby catalyzing the first step in the generation of the amyloid β (Αβ) peptide, a key pathogenic driver early in AD pathogenesis [6]. BACE1 is also proposed as a drug target for glioblastoma, where it was recently described to control macrophage phagocytosis of tumor cells [7]. BACE1 has a close homolog, BACE2, which is mostly expressed outside of the brain, at least under non-inflammatory conditions [8].

Multiple BACE1-targeted inhibitors have advanced to phase 2/3 clinical trials for AD and successfully lowered Aβ levels in brain and cerebrospinal fluid (CSF). Yet, most clinical trials were terminated early due to futility and/or the occurrence of side effects, such as non-progressive cognitive worsening and, less frequently, treatment-associated neuropsychiatric findings and sleep disturbances [9–11]. The molecular basis of these side effects is not yet clear, but needs to be elucidated to support future BACE inhibitor trials. Many side effects were common for multiple structurally-distinct BACE inhibitors that were delivered at doses that achieved > 75% inhibition of BACE activity in the central nervous system and are therefore assumed to be mechanism-based and likely to result from too strong inhibition of cleavage of one or several of the established BACE1 substrates (e.g. APP, SEZ6, CHL1) or the numerous substrate candidates, which were identified, mostly in proteomic experiments in vitro [12–25]. However, for most of these proteins it remains unclear a) whether their cleavage by BACE1 also occurs in vivo and is lowered upon pharmacological BACE1 inhibition, b) whether their cleavage products may be detected diagnostically in body fluids (CSF and blood) and may serve as prognostic safety biomarkers and c) whether their cleavage is linked to physiological functions and, thus, may potentially contribute to the side effects observed in the clinical trials.

Identification of BACE1 substrates in vivo is feasible using mouse CSF [21, 24], because the membrane protein BACE1 is highly expressed in brain, mostly in neurons, and cleaves off the ectodomain of its membrane protein substrates in a process referred to as ectodomain shedding [26]. As a result, the substrate ectodomains are shed into the extracellular space, including into CSF.

Besides mice, non-human primates (NHP), such as the rhesus monkey *Macaca mulatta*, are increasingly used as a preclinical model for the study of CNS pharmacodynamics and pharmacokinetics in the context of neurodegenerative, neuroinflammatory and neurovascular diseases such as AD [27–29], Parkinson’s disease [30], multiple sclerosis [31] and stroke [32]. Compared to mice, NHPs are more closely related to humans (93% gene sequence identity to *Homo sapiens*) [33] and allow sampling of larger blood-free CSF volumes and even repeated CSF sampling via surgical placement of a cisterna magna port [34]. Additionally, they develop naturally age-dependent AD-related amyloid pathology without transgenic approaches [35]. To date drug dosing studies in *Macaca mulatta* mostly relied on the measurements of single analytes, such as Aβ [28, 29]. Only few proteomic studies have been carried out with nonhuman primate CSF [29, 32, 36–40].

To identify in vivo relevant BACE1 substrates, we carried out a pharmacoproteomics study and demonstrate that BACE inhibition induces specific dose-related changes in the NHP CSF proteome. The strongest reductions were observed for the cleaved, soluble ectodomains of the known BACE substrates SEZ6 and VCAM1 as well as of the cytokine receptor gp130 (also known as IL-6 signal transducer (IL6ST) or Interleukin-6 receptor subunit beta), which we establish as an in vivo BACE1 substrate. Reduction of soluble gp130 (sgp130) was also observed in the CSF of participants of a clinical trial with a BACE inhibitor and even in the plasma of BACE1 KO mice, suggesting the use of sgp130 as a pharmacodynamic marker for BACE1 activity in vivo. gp130 transmits signals of the IL-6 family of cytokines and is linked to inflammation, infection and cancer, but also has essential homeostatic roles, for example in metabolism and neural development [41–43]. Mechanistically, we demonstrate that BACE1 directly cleaves gp130 and controls its signaling in primary neurons, thereby enabling neuronal survival upon growth-factor withdrawal.

## Methods

### NHP sample preparation for proteomics

Rhesus monkeys chronically implanted with catheters in the cisterna magna allowed repeated sampling of CSF in conscious animals. All animal procedures were done in accordance with guidelines from the Institutional Animal Care and Use Committee at Merck.

#### MBI-4 study

Rhesus monkeys (*Macaca mulatta*) with in-dwelling cisterna magna cannulas were administered single oral doses of the BACE inhibitor MBI-4 (10 mg/kg, 30 mg/kg and 100 mg/kg; *N* = 3/dose level) and a vehicle control (*N* = 3) (0.4% hydroxypropylmethylcellulose) as described previously [28]. CSF collected in that study at time points of − 24, − 1, 12 and 24 h relative to compound dosing was taken for MS analysis. Time points were based on the CSF Aβ and sAPPβ biomarker profiles previously described [28]. Overall, 48 samples were subjected to proteolytic digestion and LC-MS analysis. 50 μL of each CSF sample was enzymatically digested with 0.5 μg LysC and 0.5 μg trypsin using the filter assisted sample preparation (FASP) [44]. Subsequently, the proteolytic peptides were desalted by stop and go extraction (STAGE) with C18 tips [45]. The purified peptides were dried by vacuum centrifugation. Samples were dissolved in 20 μL 0.1% formic acid. Peptide yield was estimated by absorption at 280 nm using a nanoDrop photometer (Thermo Scientific, US).

#### Verubecestat study

Rhesus monkeys (*Macaca mulatta)* with in-dwelling cisterna magna cannulas were administered single oral doses of verubecestat at 1 or 3 mg/kg prepared in 0.4% Hydroxypropylmethycellulose (*N* = 3/dose level). CSF was collected at − 24, − 21 and − 2 hour followed by CSF collection at 0, 2, 6, 8, 12, 24 and 48 hours post dose. Proteomics analysis of 27 CSF samples was performed on CSF from − 24, 0, and 24 h time points. Verubecestat produced the anticipated time and dose related decreases in the levels of CSF Aβ and sAPPβ biomarkers (Fig. S1 e-g). Sample preparation and LC-MS/MS proteomics analysis were conducted as previously described with modifications [46]. Briefly, protein concentration of CSF were measured by BCA assay (Thermo Fisher Scientific) and proteins were digested using the S-trap approach (Protifi, [47]) following manufacturers protocol. The proteolytic peptides were then desalted [45] and were dried by vacuum centrifugation prior to TMT labeling to generate 3 TMT11 plex as described [48]. The labeled peptides were pooled and further desalted and dried to completion. Peptide fractionation was carried out by high-pH reverse phase chromatography following the protocol described previously [49]. In brief, 200 μg of peptides were separated by a linear gradient from 4% buffer B (100 acetonitril) / 98% buffer A (2.5 mM ammonium bicarbonate, pH = 8, in water) to 48% buffer B in 48 min followed by a wash at 85% buffer B and equilibration of column at 4% buffer B. Ninety-six fractions were collected and pooled into twenty-four fractions. Peptide fractions were frozen at − 80 °C freezer and dried to completion.

### Mass spectrometric measurements

#### MBI-4 study

Each sample was analyzed by LC-MS/MS. A peptide amount of 1 μg was separated on a nanoLC system (EASY-nLC 1000, Proxeon – part of Thermo Scientific, US) using an EASY-Spray column (50 cm × 75 μm ID, PepMap C18, 2 μm particles, 100 Å pore size, Proxeon – part of Thermo Scientific, US) with a binary gradient of water (A) and acetonitrile (B) containing 0.1% formic acid (0 min., 2% B; 5 min., 5% B; 185 min., 25% B; 230 min, 35% B; 250 min, 60% B; 255 min., 95% B; 270 min., 95% B) at 55 °C column temperature.

The nanoLC was coupled online via an Easy spray (Proxeon – part of Thermo Scientific, US) electrospray ion source to a Q-Exactive mass spectrometer. Full MS spectra were acquired at a resolution of 70,000. The top 10 peptide ions exceeding an intensity of 2.0 × 10^4^ were chosen for collision induced dissociation. Fragment ion spectra were acquired at a resolution of 17,500. A dynamic exclusion of 60 s was used for peptide fragmentation.

#### Verubecestat study

Pooled fractions from 27 TMT labeled samples were dissolved in 0.1% formic acid and an equivalent of 500 ng based on the input for the fractionation was used per measurement. Samples were subjected to an UltiMate 3000 nano LC system coupled to Orbitrap Exactive Plus mass spectrometer [48]. A non-linear gradient from 2% buffer A (0.1% formic acid, 5% DMSO in water) and 98% buffer B (0.1% formic acid, 5% DMSO in acetonitrile) to 32% buffer B in 100 min was applied. MS1 spectra were acquired at a resolution of 70,000 using an automatic gain control (AGC) target value of 1e6 with maximum injection time 50 ms. After peptide fragmentation via higher energy collisional dissociation, MS2 spectra of top 10 precursors were acquired at 17,500 resolution using an AGC target value of 5e4 and a maximum injection time of 50 ms.

### Proteomic data analysis

The data was analyzed by the software Maxquant (maxquant.org, Max-Planck Institute Munich) version 1.5.0.12 [50]. The MS data was searched against a reverse concatenated fasta database including isoforms of *Macaca mulatta* from UniProt (download: July 23rd 2014, 35,572 entries). Trypsin was defined as protease. Two missed cleavages were allowed for the database search. The option first search was used to recalibrate the peptide masses within a window of 20 ppm. For the main search peptide and peptide fragment mass tolerances were set to 4.5 and 20 ppm, respectively. Carbamidomethylation of cysteine was defined as static modification. Acetylation of the protein N-terminus as well as oxidation of methionine was set as variable modifications. The false discovery rate for both peptides and proteins was adjusted to less than 1%. Label free quantification (LFQ) of proteins required at least two ratio counts of unique peptides. Only unique peptides were used for quantification. The LFQ values for 12 and 24 h post-dose were divided by the average of the related baseline LFQ values at − 24 and − 1 h. These ratios were log_2_-transformed. Only proteins with three valid baseline ratios for MBI-4 and vehicle treatments were subjected to statistical analysis. The statistical analysis was performed with the software Perseus (version 1.6.14.00) [51]. Two-sided student’s t-test with permutation based FDR correction [52] were applied to identify significant differences of protein abundance in the CSF between the different MBI-4 doses and the related vehicle controls.

Resulting raw files from the Verubecestat study were directly analyzed in MaxQuant 1.6.2.6 with the following settings: Trypsin with up to two missed cleavages; fixed modifications: Carbamidomethyl (C); variable modifications: Oxidation (M); Acetyl (Protein N-term); protein sequence database: *Macaca mulatta* UniProt; PSM/Protein FDR: 1%.;TMT11-plex label(MS2-based labeling). Data filtering and analysis was done using Perseus software (version 1.6.14.0) and Python. Intensity values were log2 transformed and normalized against the intensities of the individual pools in each TMT plex. Cross-plex normalization was done by median centering for each protein within each plex (row-wise normalization) [53]. For statistical analysis, the individual log2 ratios versus the respective baseline values were calculated and a two-sided student’s t-test with permutation based FDR correction [52] was applied to identify significant differences 1 mpk, 3 mpk and the vehicle control.

### Determination of verubecestat, sAPPβ and Aβ concentrations

Verubecestat concentrations in rhesus monkey plasma and CSF were measured as described [54] and are reported as total values. Rhesus CSF levels of sAPPβ, Aβ1–40 and Aβ1–42 were measured using Mesoscale Discovery human immunoassays as described [28].

### Immunoblot analysis

Monoclonal antibodies against murine sSEZ6 (clone 14E5–11; IgG1) [24], human sAPPβ (clone 8C10, BAWT) were used [55]. Additionally, an antibody against human sgp130 (R&D systems, AF-228, US), human serum albumin (Abcam, A6684), mouse gp130 (Santa Cruz, sc-656, US), mouse sgp130 (R&D systems, AF-468, US), mouse sNrCAM (Abcam, ab24344), β actin (Sigma-Aldrich, A5316), mouse BACE1 3D5 (kindly provided by Dr. Robert Vassar), mouse STAT3 (Cell Signaling, clone 124H6) and mouse phospho-STAT3 (Cell Signaling, clone D3A7) were used. HRP-coupled anti-mouse and anti-rabbit secondary (DAKO, Germany), and HRP coupled anti-goat and anti-rat antibodies (Santa Cruz, US) were used. For human serum albumin, an anti-mouse secondary antibody with Alexa Fluor 488 was used (Thermo Fisher Scientific, A-11029). The following reagents and media were used: neurobasal medium, HBSS, MEM and B27 (Invitrogen), C3 (β-secretase inhibitor IV; Calbiochem, 565,788).

For NHP CSF samples, baseline samples (− 24 h) as well as 12 h and 24 h post dose samples of each experiment (vehicle, 10 mg/kg, 30 mg/kg and 100 mg/kg BACE inhibitor) were separated on 8% SDS-polyacrylamide gels. A volume of 15 μL CSF was used per lane. Samples for sAPPβ and sSEZ6 detection were boiled for 5 min at 95 °C in Laemmli buffer. For sgp130, Laemmli buffer without β-mercaptoethanol was used. Proteins were transferred to a nitrocellulose membrane with a tank blotting system (Biorad, Germany). The membranes were blocked in 6% nonfat dried milk in PBS-T (Phosphate-buffered saline, pH 7.3, and 0.05% Tween 20) for 1 h and washed in PBS-T. Incubation with primary antibody was done for 1–2 h at room temperature or at 4 °C overnight. Membranes were incubated in secondary antibody (anti-rat for sSEZ6, anti-mouse for sAPPβ, anti-goat for sgp130) at room temperature for 1 h after washing with PBS-T. Human CSF samples of single dose BACE inhibition (baseline: 0 h and post dose: 30 h) were blotted against sSEZ6 as described above. For serum albumin, blots for NHP CSF were stripped in a commercial stripping buffer (Thermo Fisher Scientific, 46,430) for 15 min and developed again with a human serum albumin antibody and a secondary fluorescent antibody using an iBright FL 1500 imager (Thermo Fisher Scientific, US).

For cell lysates and supernatants of murine primary cell culture and HEK293T (ATCC, US) cell culture, 20 μg of total protein was separated by SDS–PAGE on 8% Tris-Glycine gels and transferred onto PVDF membranes. The membranes were blocked in 5% milk prepared in TBS-0.1% Tween and incubated in primary antibodies overnight at 4 °C followed by washing and incubation with secondary antibody. Blots were developed using the ImageQuant LAS 4000 mini machine (GE Healthcare) and band intensities were quantified with ImageJ analyzer software (1.49v). The levels of the full-length (FL) proteins were normalized to actin levels.

Quantification of immunoblots was performed with the software ImageJ (1.49v) according to the recommendations of the NIH (imagej.nih.gov) [56]. For NHP CSF samples, the intensities were normalized to their corresponding baseline values and log2 transformed. The statistical analysis was performed with the software GraphPad Prism. For NHP CSF, a two-way ANOVA test was used to determine significant differences between the time points and the dosing groups (10 mg/kg vs. vehicle, 30 mg/kg vs. vehicle, 100 mg/kg vs. vehicle) applying a Dunnett’s multiple comparisons posthoc test. For cell lysates and supernatants, immunoblot band intensity was normalized to that of a vehicle-treated sample of each independent experiment and paired t test was used to determine statistical differences.

### Verubecestat phase 1 clinical trial samples

Human CSF was derived from a single ascending dose Phase 1 study of verubecestat in normal healthy volunteers. The human clinical study was conducted in accordance with International Conference on Harmonisation Good Clinical Practice guidelines and was approved by the relevant institutional review boards. Written informed consent was provided by the patients or their legal representatives. The full details of the study and study protocol were previously published [27].

### BACE1 in vitro cleavage assay

HEK293T cell were transfected with pcDNA3.1_Zeo(+)CD5(signal peptide)-HA-gp130-HIS. Recombinant protein HA-gp130-HIS was purified from lysates of stably expressing HEK293T cells using anti-HA-agarose (Sigma). The beads were incubated with recombinant mouse BACE1 (R&D Systems, Minneapolis, MN) in 50 mM sodium acetate buffer pH 4.4 for 16 h. Incubation of BACE1 was done with or without BACE inhibitor, C3. The samples were boiled in reducing Laemmli buffer and applied to Western blot analysis as described above.

### Isolation and culture of primary neurons and glia

Wild-type and BACE1 conditional knock-out mice used for preparation of primary neurons and glial culture were obtained from The Jackson Laboratory (B6.129-Bace1tm1Pcw/J). All animal experiments were performed according to the European community council directive (86/609/ECC). Neurons were isolated as previously described at E15/E16 and cultured in neurobasal medium supplemented with 2% B27, 100 U/ml penicillin, 100 μg/ml streptomycin and 0.5 mM glutamine [57]. Experiments were carried out after 6–7 days in vitro (DIV). Glia cultures were prepared from E16 embryos. The cortices were cleared from meninges, cut into small pieces, digested by trypsin, and filtered through a 70-μm filter. Glia cultures were maintained in MEM with Earle’s salt and 0.5 mM glutamine, 12.5% FBS, 0.6% glucose, 100 U/ml penicillin, 100 μg/ml streptomycin. Experiments were carried out after two splitting cycles. Cell lysates were prepared in 50 mM Tris-HCl, pH 7.4, 150 mM NaCl, 2 mM EDTA, and 1% Triton X-100 supplemented with complete protease inhibitor (Roche Applied Science). Cells were lysed for 30 min on ice and cleared by centrifugation at 12,000 g for 10 min. Protein concentrations were measured using standard BCA assay (Pierce).

### Transduction of cells with lentiviruses encoding shRNAs or CRE

Codon-improved Cre recombinase (iCre) lentiviruses were prepared as previously described [58]. The following shRNA targeting gp130 sequence were used: shRNA1: 5′CGCGTCCGGCTTGCCCAGGCAACCGTATTTCTCGAGAAATACGGTTGCCTGGGCAAGTTTTTGGAAA′3; shRNA2: 5′CGCGTCCGGCAAAGTGTGTCTGAGTTTATACTCGAGTATAAACTCAGACACACTTTGTTTTTGGAAA′3. Targeting sequences, as well as scrambled control sequence, were cloned into plKO2mod-EGFP-WPRE, as previously described [58]. Lentiviruses were generated by transient cotransfection of HEK293T cells with the plasmids psPAX2, pCDNA3.1-VSV-G and transfer vector F2UΔZeo-iCre or shRNA vector plKO2mod-EGFP-WPRE using Lipofectamine 2000 (Thermo). Lentiviral particles for infection of murine primary cortical neurons were concentrated and purified by ultracentrifugation. Lentiviral stocks were stored at − 80 °C until use. Alternatively, fresh virion particles were used for infection of murine primary cortical neurons.

### Elisa

For human CSF, the human soluble gp130 Quantikine ELISA Kit (DGP00, R&Dsystems, US) was used. CSF samples were thawed and diluted 1:20 in Calibrator diluent from the kit. Afterwards the samples were run in the sgp130 Elisa kit from R&D according to the manufacturer’s instructions for tissue culture supernatants (2 h of incubation for the conjugate).

For cell culture samples, conditioned medium of 7 million cells was collected and filtered through 0.45 μm PVDF filter (Millex) into a VivaSpin 20 column (30 kDa) at 4 °C. Proteins were concentrated to 300 μl and applied to IL6ST/gp130 Mouse ELISA Kit (Invitrogen, EMIL6ST). Experiment was performed by manufacture procedure. Gp130 concentration was calculated by standard curve corresponding to gp130 concentration. ELISAs were measured using a Tecan M200 infinite pro microplate reader (Tecan, Austria) and analyzed with the Magellan software (V 7.2, Tecan, Austria).

### Mouse serum and plasma analysis

Blood of mice was collected immediately after decapitation. For obtaining serum from adult BACE−/− mice [59] and control mice, blood was incubated at room temperature for 30 min. Serum was collected after centrifugation at 2000 x g for 15 min. For obtaining plasma, blood of BACE1−/− mice [60] and control mice at P5 was collected immediately into EDTA-coated collection tubes. After centrifugation step at 6000 x g for 5 min, sgp130 was measured using an ELISA kit (DY468, R&D systems) according to manufacturer’s instructions.

### Analysis of gp130 shedding in neurons and glial cells

To enrich endogenous sgp130 from the conditioned medium of neurons or glial cells, we used the secretome protein enrichment with click sugars (SPECS) method [12, 14]. For each condition, 1.5 million 4 DIV neurons or 1 million glial cells were labelled with 1 μmol of tetraacetyl-N-azidoacetyl-mannosamine diluted in 20 ml relevant medium (50 μmol/l) supplemented with either 2% B27 or 10% FBS for 2 days. Cells were cultured in the presence or absence of 2 μM BACE1 inhibitor C3 or DMSO as a control.

Conditioned medium was collected and filtered through 0.45 μm PVDF filter (Millex) into a VivaSpin 20 column (30 kDa) at 4 °C. To remove non-metabolized tetraacetyl-N-azidoacetyl-mannosamine VivaSpin 20 columns were centrifuged at 2000 g at 4 °C. The retentate was filled with 20 ml PBS. This procedure was repeated three times. In the last step, the PBS refill step was omitted. Instead, 250 nM of Sulfo-DBCO-biotin (Click-chemistry tools) diluted in 1 ml PBS was added to retentate to biotinylate metabolically azide-labelled glycoproteins. Columns were incubated overnight at 4 °C. For removal of non-reacted Sulfo-DBCO-biotin, VivaSpin20 columns were subject to three times of centrifugation with subsequent PBS buffer refill. For purification of biotinylated proteins, the sample was incubated with streptavidin magnetic beads (Sigma-Aldrich). After binding of proteins, streptavidin beads were washed with PBS with 1% SDS. Afterwards, streptavidin beads were boiled with urea sample buffer containing 3 mM biotin to compete for the binding of biotinylated proteins with streptavidin.

### sGp130 purification

HEK293T cells were transfected with pcDNA3.1 Zeo(+)CD5-HA-SLIC-HIS-sgp130 for 48 h. The plasmid encodes the CD5 signal peptide, followed by an HA tag, a SGAGGSSD linker, a His-tag, a thrombin cleavage site and then the murine gp130 ectodomain (Uniprot ID Q00560) comprising amino acids 23–617 (where the transmembrane domain starts). Conditioned medium was collected, filtered, adjusted to pH 7.5 and supplemented with 5 mM imidazole, 1 mM DTT and complete protease inhibitor (Roche Applied Science). His-tagged sgp130 was purified on a 5 mL Ni/HisTrap HP column (GE healthcare, Germany). Thrombin protease was used to elute sgp130 into PBS buffer. Thrombin was removed using 1 mL HiTrap Benzamidine column (GE healthcare, Germany). Purity of the protein was assessed by SDS-PAGE and mass spectrometry to 95%.

### Analysis of gp130 signaling

Neurons at DIV 7 were treated with 10 ng/ml recombinant mouse IL6/IL6Rα complex (R&D systems, 9038-SR, US) for 15 min in the presence or absence of 50 ng/ml mouse sgp130, which was purified from HEK293T cells. The reaction was stopped with ice-cold rinsing buffer (20 mmol/L Tris-HCl (pH 7.6) buffer containing 138 mmol/L NaCl. Cell lysates were prepared in RIPA buffer (50 mmol/L Tris-HCl (pH 7.4) containing 0.15 mol/L NaCl, 0.25%(w/v) sodium deoxycholate, 1.0%(v/v) Nonidet P-40, 1.0 mmol/L EDTA, 1.0 mmol/L phenylmethanesulfonyl fluoride, 1.0 mmol/L Na_3_VO_4_, 1.0 mmol/L NaF and complete protease inhibitor (Roche Applied Science). Cells were lysed for 30 min on ice and cleared by centrifugation at 10,000 g for 10 min. Protein concentrations were measured using standard BCA assay (Pierce).

### Neuronal MTT

Neurons at DIV4 were cultured in neurobasal medium without B27, and treated with or without C3, following by activated by 10 ng/ml H-IL-6. For rescue experiments 50 ng/ml sgp130 were added. At DIV12, neuronal survivability was evaluated by MTT kit according to manufacture’s procedure (CyQUANT™ MTT Cell Proliferation Assay Kit, ThermoFisher, V13154).

### Statistics

Statistical analysis was done using GraphPad Prism and Perseus [51] software.

## Results

### BACE inhibition specifically alters the composition of the non-human primate CSF proteome

**Fig. 1:**
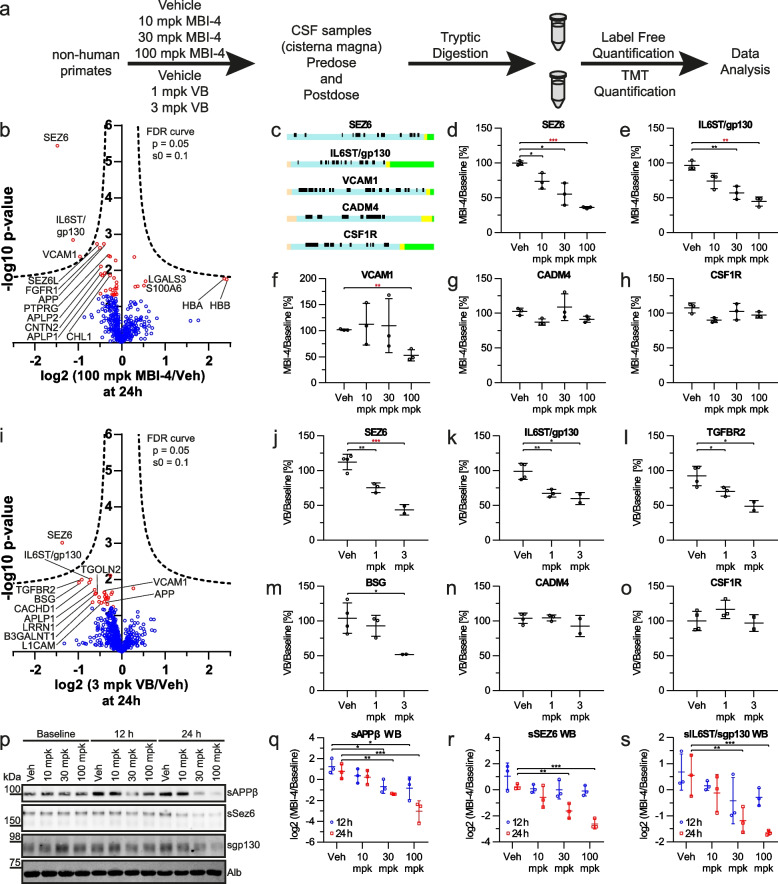
Proteomic analysis of non-human primate CSF. **a** Workflow of proteomic CSF analysis after single-dose BACE inhibition using either label free or tandem mass tag (TMT)-based quantification. **b** Volcano plot of BACE inhibition with 100 mg/kg (mpk) MBI-4 at 24 h (*N* = 3 per condition). The log2 transformed ratios are plotted against the log10 transformed *p*-value of the Student’s t-test. (red: proteins with *p* < 0.05; dashed line: FDR threshold for multiple hypotheses; *N* = 3). **c** Peptide mapping onto the topology of SEZ6, IL6ST, VCAM1, CADM4, and CSF1R using the web-based software tool QARIP [61] shows that the identified peptides are solely derived from the protein ectodomains (pink: signal peptide, blue: ectodomains yellow: transmembrane domains, green: cytoplasmic domains, black: identified peptides) **d-f** Dot plots for the relative MS quantification of the BACE1 substrate SEZ6 (**d**), the BACE1 substrate candidate IL6ST/gp130 (**e**) and the BACE2 substrate VCAM1 (**f**) at 24 h. **g-h** As controls, dot plots of the ADAM10 substrate CADM4 (**g**) and the ADAM17 substrate CSF1R (**h**) are shown. **i** Volcano plot of BACE inhibition with 3 mpk verubecestat (VB) at 24 h (vehicle *N* = 4; 3 mpk *N* = 2). **j-o** Dot plots for the MS quantification of the BACE1 substrate SEZ6 (**j**), the BACE1 substrate candidates IL6ST/gp130 (**k**) and TGFBR2 (**l**) as well as BSG (**m**), CADM4 (**n**), and CSF1R (**o**). (*: *p* < 0.05; ** *p* < 0.01; *** *p* < 0.001; red asterisks indicate significance after permutation-based FDR correction). **p** Western blots against shed APPβ, sSEZ6, and sgp130 in NHP CSF and against albumin (Alb) as a loading control (*N* = 3). **q-s** Dot plots for the quantification of Western blots in (**p**) based on the CSF of three individual NHPs at 12 (blue) and 24 h (red) post application. Shown are the mean log2 ratios of MBI-4 or Vehicle (Veh) versus the respective baseline values ± SD; For immunoblot quantification a two-way Anova test for time and dose with a Dunnett’s multiple comparisons posthoc test comparing the vehicle with the BACE inhibitor treatments was applied.*: *p* < 0.05; **: *p* < 0.01; ***: *p* < 0.001

The CSF of three individual NHPs (*Macaca mulatta*) treated in a previous study [28] with a single oral dose of the BACE inhibitor MBI-4 at 10, 30, or 100 mg/kg or vehicle was used for pharmacoproteomics. Similar to BACE inhibitors used in clinical trials, MBI-4 inhibits both BACE1 (BACE1 K_i_ = 4.7 nM) and its homolog BACE2 (BACE2 K_i_ = 2.7 nM) [28] and achieved CSF concentrations of 0.9, 4.6, and 23.4 nM in NHPs with a single oral dose of 10, 30, or 100 mg/kg, respectively [28]. For proteomics, two pre-dose CSF samples taken at − 24 h and − 1 h were used to define baseline proteome profiles. Post-dosing samples were taken from the 12 h and 24 h time points, when BACE1 is maximally inhibited, based on the reduction of the BACE1-mediated APP-derived fragments Aβ and sAPPβ, as previously monitored in the same samples [28]. The CSF samples were proteolytically digested and resulting peptides were analyzed by liquid chromatography-mass spectrometry (LC-MS)-based shotgun proteomics using label-free quantification (LFQ) (Fig. 1a).

In the NHP CSF, 983 proteins were identified by at least two unique peptides (Fig. 1b), including proteins with key roles in neurological diseases, such as APP, TREM2 and ApoE in AD [6], DJ-1/PARK7 in Parkinson’s disease [62] and the prion protein in prion diseases [63]. After individual baseline normalization to the pre-dose LFQ intensities, we relatively quantified 580 and 598 proteins in all vehicle and BACE inhibitor-treated NHP samples at 12 and 24 h, respectively (Fig. 1b, Fig. S1a). Similar to murine and human CSF [21], most proteins were annotated as secreted (329; 53%) or membrane (249; 40%) proteins (Fig. S1b). The correlation of the median log10 transformed iBAQ intensities showed an excellent correlation between the human and NHP CSF proteome with a Pearson correlation of 0.86. Within each species the correlation was even larger than 0.97 (Fig. S1c, d). The identified peptides were mapped onto the topology of the corresponding proteins with the web-based tool QARIP [61]. The peptides of 94% (118/126) of transmembrane type 1, 100% (34/34) of transmembrane type 2, 100% (12/12) of multipass transmembrane as well as 97% (35/36) of GPI-anchored proteins exclusively matched to the protein ectodomains (Suppl. Tab. 1, 2 and 3 and shown for selected proteins in Fig. 1c), indicating that those CSF proteins do not constitute the full-length proteins, but are derived from proteolytic ectodomain shedding [26].

Compared to the vehicle group, the single dose of 100 mg/kg (mpk) MBI-4 significantly reduced the CSF abundance of the ectodomain of three proteins (SEZ6, IL6ST/gp130, VCAM1) to 25–50% at 24 h post-dosing when applying false discovery rate correction [52] (Fig. 1b, Supplementary Data 1). SEZ6 is a known BACE1 substrate [12, 23, 24, 64], whereas VCAM1 is a substrate for BACE2 in glial cells [8], which do not express BACE1. IL6ST is the cytokine receptor gp130 and was previously suggested as a potential BACE1 substrate candidate in two proteomic studies using non-neuronal cell lines [13, 15], but was not further studied. Several additional membrane proteins showed reduced ectodomain abundance, including the known BACE1 substrates SEZ6L, APP, APLP1, APLP2, CNTN2 and CHL1 [12, 24, 25], but had a lower fold-change compared to SEZ6, gp130 and VCAM1. Additional membrane proteins with reduced ectodomain abundance in CSF were FGFR1 and PTPRG (Suppl. Data 1), which we consider as new BACE1 substrate candidates. The peptides identified for all of these proteins derived only from their ectodomains but not their transmembrane or cytoplasmic domains (Suppl. Tables 1, 2 and 3), which is in line with their known or presumed generation through BACE1 cleavage.

For APP, APLP2, CNTN2, VCAM1, IL6ST/gp130, and SEZ6 the reduction occurred in a dose-dependent manner (Fig. 1d-f, Fig. S2). The extent of ectodomain reduction differed for the individual proteins following the single dose, with sSEZ6 and sgp130 showing the strongest reductions. Possibly, both proteins have a short half-life in CSF and, thus, may be considered as rapid response substrates when BACE1 activity changes acutely, whereas repeated inhibitor dosing to achieve stable levels of inhibitor exposure and BACE inhibition would be required to more effectively lower CSF abundance of the other proteins. For example, SEZ6L showed a similar reduction as SEZ6 in the CSF of BACE1/2 double KO mice [24], but showed a weaker decrease with acute inhibition of BACE1 (Suppl. Data 1, Fig. S2), potentially because of a longer half-life. Alternatively, the substrates besides SEZ6 and gp130 may additionally be cleaved by proteases other than BACE1, so that BACE1 inhibition would only partly reduce their CSF ectodomain abundance. As a control, the ectodomains of CADM4 and CSF1R, which are cleaved by ADAM10 and ADAM17, respectively [65, 66], were not reduced (Fig. 1g, h).

Besides BACE1 substrate ectodomains being reduced in CSF, MBI-4 treatment also increased CSF abundance of a few proteins, but only the 5.3-fold increase of hemoglobin subunits α and β (HBA, HBB) came close to reaching statistical significance upon multiple hypothesis testing (Fig. 1b, Fig. S2q, r). A blood contamination during sampling is possible, but appears unlikely, because even a minor blood contamination results in dramatic changes in the CSF proteome [67], which, however, was not observed. As soluble proteins reported to be expressed in neurons of the mammalian CNS [68], the increase in HBA and HBB may be a secondary response to acute BACE inhibition by MBI-4 or be a structure-related off-target effect.

To validate the results obtained with MBI-4, we repeated the pharmacoproteomic CSF analysis with an independent cohort of NHPs (*macacca mulatta*), but with a distinct BACE inhibitor – the clinically tested verubecestat [27] – and employing a different proteomic method based on tandem mass tag (TMT)-based relative quantification. NHPs were treated with a single dose of vehicle (4 animals), 1 mg/kg (3 animals) or 3 mg/kg (2 animals). The 3 mg/kg dose was reported in a previous study to lower CSF Aβ40 by a peak 76% at 12–24 hours [27] and this was also seen in the current study (Fig. S3). Pre-dose baseline CSF samples were taken at − 24 h and 0 h. A post-dosing CSF sample was taken at 24 h. Overall, 1067 and 1046 proteins were relatively quantified comparing 1 mg/kg versus placebo and 3 mg/kg versus placebo, respectively. Several known or proposed BACE1 substrates such as SEZ6, CACHD1, LRRN1, L1CAM, and gp130/IL6ST showed a dose-dependent reduction in CSF, with the reduction of SEZ6 remaining significantly changed after FDR correction (Fig. 1i-k, Suppl. Data 2). Additionally, the single-span transmembrane proteins BSG, TGOLN2 and TGFBR2 displayed dose-dependent reductions (Fig. 1l-m, Suppl. Data 2), and therefore represent novel BACE substrate candidates. Similar to MBI-4, verubecestat did not affect CSF abundance of the ADAM protease substrates CADM4 and CSF1R (Fig. 1n, o). In contrast to MBI-4, verubecestat did not increase HBA and HBB (Fig. 1i, Suppl. Data 2), demonstrating that the effect of MBI-4 on both hemoglobins is not a general effect of BACE1 and/or BACE2 inhibition. Taken both proteomic studies together, we conclude that BACE inhibition in NHPs induces a specific, dose-dependent proteomic fingerprint in CSF (Table 1), consisting mostly of reduced abundance of BACE1 and BACE2 substrate ectodomains. Additionally, both studies identify IL6ST/gp130 as a novel BACE1 substrate candidate.

**Table 1:** Substrates and substrate candidates of BACE1 identified by NHP pharmacoproteomics^a^

UniProt AC	Gene	Protein Name	log2 fold change (100 mpk MBI-4/Vehicle)	log2 fold change (3 mpk MBI-4/Vehicle)	P (100 mpk MBI-4/Vehicle)	P (3 mpk MBI-4/Vehicle)
A0A1D5QZV9	APLP1	Amyloid-like protein 1	−0.39	− 0.62	1.20E-02	1.97E-02
F7FJ90	APLP2	Amyloid-like protein 2	−0.48	−0.40	7.79E-03	2.36E-02
F7ELT5	APP	Amyloid beta A4 protein	−0.41	−0.52	1.85E-03	3.07E-02
F6Y3S7	BSG	Basigin	NaN	−0.98	NaN	1.22E-02
F7B8S4	CHL1	Neural cell adhesion molecule L1-like protein	−0.27	−0.31	1.37E-02	9.60E-02
F6ZIK5	CNTN2	Contactin-2	−0.48	−0.36	1.26E-02	3.30E-02
F7A4T4	FGFR1	Fibroblast growth factor receptor 1	−0.50	−0.23	2.40E-03	3.64E-01
F7FXB6	IL6ST	Interleukin-6 receptor subunit beta	−1.12	−0.72	1.45E-03	1.00E-02
A0A5F7ZLI9	L1CAM	Neural cell adhesion molecule L1	−0.20	− 0.47	3.32E-01	4.45E-02
F6R6D9	LRRN1	Leucine-rich repeat neuronal protein 1	−0.53	−0.61	6.12E-02	2.54E-02
F7DWH2	PTPRD	Receptor-type tyrosine-protein phosphatase delta	−0.30	−0.18	4.02E-03	1.39E-01
F7EHH7	PTPRG	Receptor-type tyrosine-protein phosphatase gamma	−0.27	−0.25	4.15E-03	6.42E-02
F7F6J3	SEZ6	Seizure protein 6 homolog	−1.48	−1.37	3.64E-06	9.83E-04
F7GPP8	SEZ6L	Seizure 6-like protein	−0.57	−0.39	1.87E-03	3.02E-02
F6Q1B1	TGFBR2	TGF-beta receptor type-2	NaN	−0.92	NaN	1.02E-02
P60030	TGOLN2	Trans-Golgi network integral membrane protein 2	−0.37	−0.59	3.72E-01	2.43E-02
F6W7X9	VCAM1	Vascular cell adhesion protein 1	−0.96	−0.40	4.20E-03	2.29E-02

^a^The log2 fold changes and *p* values are from the volcano plot calculations in Fig. 1 and relate to the time point at 24 h post dosing. NaN: not detected and quantified

### Validation of MS-results by immunoblotting

We further validated the LC-MS-based quantification results of soluble SEZ6 (sSEZ6) and gp130 (sgp130) ectodomains with immunoblots (Fig. 1p). As a control, we also analyzed the BACE1 cleavage product of APP, sAPPβ (Fig. 1p, q). In the vehicle control, sAPPβ increased by 70% compared to the baseline value before dosing (corresponding to the zero value on the y axis), which is in line with a similar increase measured by ELISA in the same samples before [28] and may be due the repeated CSF sampling. At 24 h post dosing, sAPPβ was reduced by 62% (30 mg/kg) and 88% (100 mg/kg) compared to the baseline value (Fig. 1q), demonstrating efficient BACE1 inhibition. Shed sSEZ6 and sgp130 showed similar time- and dose response curves as sAPPβ, reaching average reductions of 84% (sSEZ6) and 68% (sgp130) at 100 mg/kg at 24 h compared to the baseline (Fig. 1r, s), similar to the results of the mass spectrometric measurements where a reduction of 64% for sSEZ6 and 55% for sgp130 had been observed in comparison to baseline (Fig. 1d, e).

### Verubecestat lowers sSEZ6 and sgp130 in human CSF

**Fig. 2:**
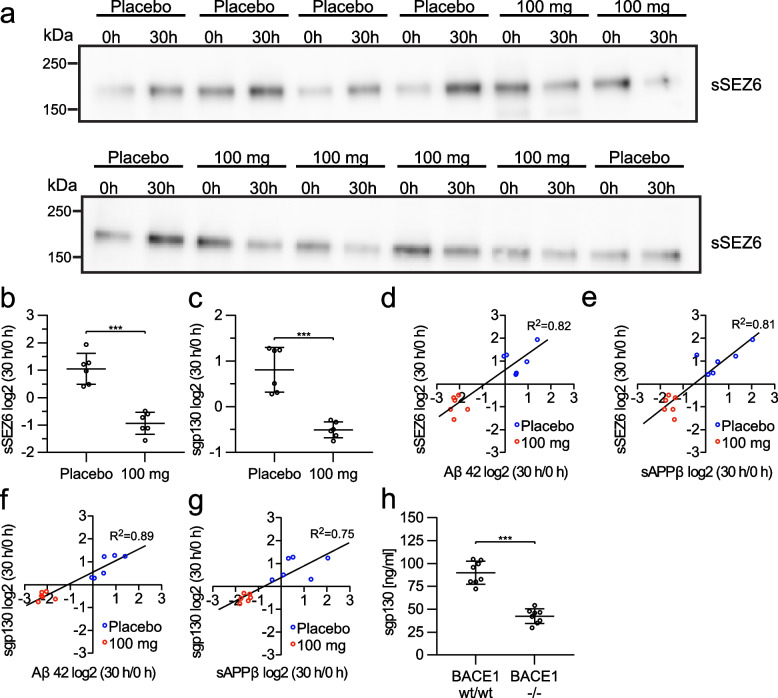
The abundance of sSEZ6 and sgp130 in body fluids is dependent on BACE1 activity. **a** Western Blots of CSF sSEZ6 from participants of a clinical phase 1 study treated with placebo or 100 mg verubecestat (*N* = 6 per condition). A volume of 15 μL of CSF was loaded per lane. The baseline samples (0 h) and post-dose samples at 30 h are plotted next to each other. **b** The quantification of the relative abundance change of sSEZ6 shows an increase of more than two-fold in the placebo group, whereas the abundance was reduced to 54% in the 100 mg verubecestat group. **c** Relative abundance change of sgp130 in the same samples measured by ELISA (*n* = 6 per condition) shows an increase to 184% in the placebo group, whereas values decrease to 71% in the 100 mg verubecestat group. **d-g** The log2 transformed post-dose to pre-dose ratios of sSEZ6 and sgp130 correlate with those detected for Aβ42 and sAPPβ (extracted from [27]). **h** The levels of sgp130 detected by ELISA in the serum of BACE1−/− mice [59] show a decrease compared to the wildtype controls (*N* = 8 per condition). Shown are mean values ± SD. Unpaired Student t-tests were performed for the individual comparisons. ***: *p* < 0.001

To test whether the observed reduction of sSEZ6 and sgp130 in CSF upon BACE inhibition can be translated to humans, we measured sSEZ6 by immunoblot (Fig. 2a, b, Fig. S4) and sgp130 by ELISA (Fig. 2c) in CSF from healthy nonelderly adults directly before dosing and 30 h after oral administration of placebo or a single dose of 100 mg verubecestat [27]. In accordance with the previously published values for Aβ and sAPPβ from the same samples [27], which showed increased Aβ40 (181%), Aβ42 (156%), and sAPPβ (187%) in the placebo group, we also observed higher post- to pre-dose ratios for sSEZ6 (221%) and sgp130 (184%) in the placebo group. Presumably, this is a result of the repeated CSF sampling (every 2 h) and/or diurnal variation [69] in each patient. In contrast, verubecestat treatment reduced CSF abundance of sSEZ6 and sgp130 to 54 and 71% of pre-dose value, which corresponds to a reduction to 24 and 39%, respectively, compared to the placebo control. These reductions are less pronounced than those detected for sAPPβ (30 h/0 h ratio: 34%) and Aβ42 (30 h/0 h ratio: 23%), but significantly correlate with each other (Fig. 2d-g). The protein abundance reduction of sSEZ6 and sgp130 in CSF detected by mass spectrometry, immunoblotting, and ELISA also showed consistent results between human and NHP as well as between different techniques (Table 2).

**Table 2:** Comparison of substrate changes between non-human-primates and humans. Protein ratio of BACE inhibition versus placebo/vehicle of sSEZ6, sIL6ST/sgp130, and sAPPβ detected by mass spectrometry (MS), Western Blotting (WB) or immunoassay ± standard deviation in %

Species	***Macaca mulatta***			***Homo sapiens***
**Inhibitor**	100 mpk MBI-4		3 mpk Verubecestat	100 mg Verubecestat
**Method**	MS^a^	WB/immunoassay	MS^a^	WB/immunoassay
**sSEZ6**	35.9 ± 1.38%	14.0 ± 4.8%^b^	38.7 ± 7.7%	25.3 ± 14.2%^d^
**sIL6ST/sgp130**	46.1 ± 6.7%	21.8 ± 2.0%^b^	60.1 ± 8.6%	40.2 ± 8.5%^e^
**sAPPβ**	–	7.1 ± 7.4%^b^ / 18.4 ± 4.8%^c^	–	21.7 ± 4.7%^f^

^a^mass spec data are taken from Fig. 1

^b^Western Blotting data are taken from Fig. 1

^c^immunoassay data for sAPPβ are taken from [27] for non-human primates

^d^Western Blotting data are taken from Fig. 2

^e^ELISA data are taken from Fig. 2

^f^immunoassay data for sAPPβ are taken from [28] for humans

In conclusion, BACE inhibitor-mediated reduction of CSF sSEZ6 and sgp130 happens similarly in NHPs and humans, suggesting a use of sSEZ6 and sgp130 as evolutionarily conserved pharmacodynamic activity markers of BACE1.

### BACE1 controls sgp130 in serum and plasma

Soluble gp130 is also detectable in human blood [70], which is more easily accessible than CSF. Thus, we tested whether plasma sgp130 levels also depend on BACE1. In fact, the sgp130 concentration was reduced by more than 50% in serum of a BACE1-deficient mouse line (Fig. 2h) [59]. The remaining sgp130 may either represent a soluble splice form of gp130, which is known to be found in body fluids [70, 71] or are generated by proteases other than BACE1. We conclude that, similar to CSF, sgp130 concentration in plasma depends on BACE1 activity. Suitable plasma from verubecestat Phase 1 studies or the NHP studies was not available for testing sgp130 levels.

### gp130 is a BACE1 substrate in vitro

**Fig. 3:**
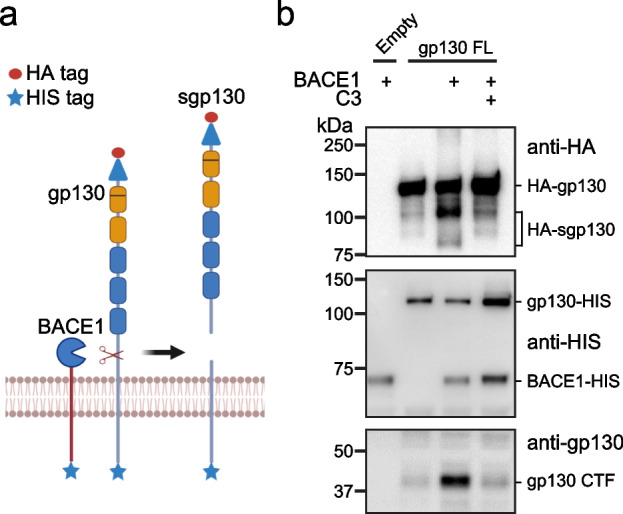
Cleavage of gp130 by BACE1 in vitro. **a** Scheme illustrating domain structure of gp130 and its proteolytic cleavage by BACE1, leading to sgp130 secretion. The Ig-like domain (blue triangle) and the five fibronectin type III domains (orange and blue) in the gp130 ectodomain are indicated. **b** In vitro cleavage assay of purified gp130 with BACE1 in the presence or absence of the BACE inhibitor C3. Western blot detects HA tag at the N-terminus of gp130 or the HIS tag at the C-terminus of gp130 and the His-tag of BACE1. For the lowest panel an antibody to the natural C-terminus of gp130 was used. Shown are representative blots for *N* = 3 independent experiments

Previously, sgp130 was assumed to be predominantly generated as a soluble protein through alternative splicing of the gp130 mRNA [70, 71]. However, our CSF and plasma analyses suggest that a large fraction of about 50% of sgp130 is instead generated through BACE1-mediated shedding of the full-length, transmembrane form of gp130. To test whether BACE1 directly cleaves gp130, we used an in vitro protease assay, in which recombinant BACE1 was incubated with full-length, membrane-bound gp130 carrying an N-terminal HA-epitope tag and a C-terminal HIS-tag (Fig. 3a). Full-length gp130 was detected at its molecular weight of about 130 kDa with antibodies to both the HA- and the HIS-tag (Fig. 3b). Addition of BACE1 led to the appearance of a smaller molecular weight band just below 100 kDa and a fainter band at around 85 kDa. Both bands were only detected with an antibody directed against the HA-, but not the HIS-tag. Thus, these fragments correspond to N-terminal gp130 cleavage products. BACE1 cleavage also generated a gp130 fragment that was detected with an antibody against the C-terminal HIS-tag. Its apparent molecular weight of about 40 kDa corresponds to a C-terminal fragment comprising the cytoplasmic and transmembrane domains and a short part of the ectodomain, consistent with BACE1 cleaving within the extracellular juxtamembrane domain of gp130 (Fig. 3b). Both the N- and C-terminal fragments were strongly reduced upon addition of the established BACE1 inhibitor C3 (also known as BACE inhibitor IV) [72]. From these experiments, we conclude that BACE1 can directly cleave gp130.

### BACE1 cleavage of gp130 happens physiologically in primary neurons

**Fig. 4:**
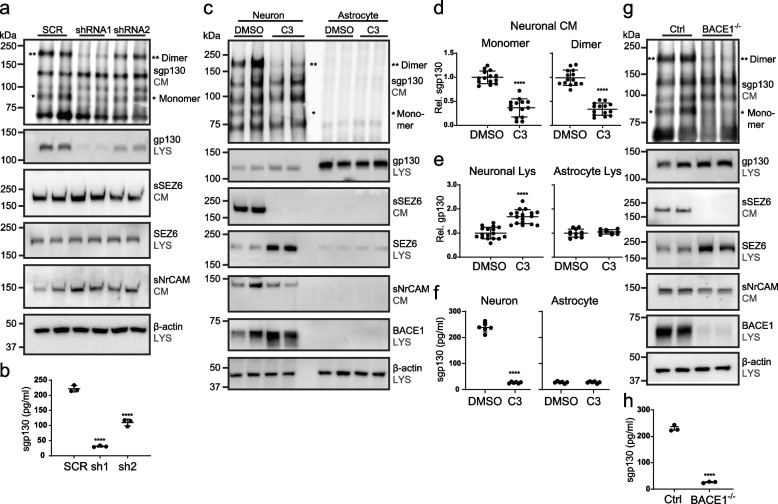
BACE1 cleaves gp130 in neurons. **a** Immunoblots of conditioned media (CM) and lysates (LYS) of primary neurons transduced with two different gp130 shRNAs or a scrambled (SCR) shRNA as control. Sgp130 was pulled-down from the conditioned medium. Full-length gp130 and SEZ6 in the lysates as well as secreted soluble gp130 (sgp130) and soluble SEZ6 (sSEZ6) were detected. Actin and sNrCAM serve as loading controls. The monomer and dimer bands of sgp130 are indicated. **b** ELISA to detect sgp130 in concentrated conditioned neuronal medium. Shown are mean ± SD from *N* = 3 biological replicates. One-way ANOVA with Dunnett’s multiple comparisons test. **c** Immunoblots against indicated proteins in conditioned media and lysates of neurons and astrocytes in the absence (DMSO) or presence (C3) of the BACE1 inhibitor C3. Actin and sNrCAM serve as loading controls. **d-e** Quantification of sgp130 monomer and dimer bands in the neuronal medium (*N* = 14 biological replicates), as well as full-length gp130 in neuronal (*N* = 17 biological replicates) and astrocyte lysates (*N* = 11 biological replicates) from immunoblots in **c**. Shown are mean ± SD. Unpaired Student t-test. **f** ELISA measurement of sgp130 in the concentrated conditioned medium from experiments in **c**. Shown are mean ± SD from *N* = 6 biological replicates. Unpaired Student t-test. **g** Immunoblots of conditioned media (CM) and lysates (LYS) of primary, floxed BACE1 neurons transduced with a control lentivirus (Ctrl) or a Cre-expressing lentivirus (BACE1^−/−^). Sgp130 was pulled-down from the conditioned medium. Full-length gp130 and SEZ6 in the lysates as well as secreted soluble gp130 (sgp130) and soluble SEZ6 (sSEZ6) were detected. Actin and sNrCAM serve as loading controls. The monomer and dimer bands of sgp130 are indicated. **h** ELISA measurement of sgp130 in the concentrated conditioned medium from experiments in **g**. Shown are mean ± SD from *N* = 3 biological replicates. Unpaired Student t-test. ***: *p* < 0.001; ****: *p* < 0.0001

To test whether BACE1 is also required for gp130 cleavage in primary cells, we turned to primary murine cerebral neurons, where BACE1 is highly expressed [2, 73, 74]. sgp130 was detected in the conditioned medium as a monomer with a band of about 85 kDa and a presumed dimer with a band of about 170 kDa. Both bands were specific to sgp130, because they were strongly reduced, when the endogenous gp130 was knocked-down using lentiviral shRNAs (Fig. 4a and b). Importantly, full-length gp130 was enriched and sgp130 levels were strongly reduced when BACE1 was blocked pharmacologically with the BACE1 inhibitor C3 (Fig. 4c-f). As a positive control, C3 in neurons also blocked shedding of the known BACE1 substrate SEZ6 [12], while it did not block secretion of the ADAM10 substrate sNrCAM, which served as a negative control [65, 75] (Fig. 4c). Similar results were obtained when BACE1 was genetically blocked using floxed BACE1 KO neurons transduced with a Cre recombinase-expressing lentivirus (Fig. 4g and h).

gp130 is ubiquitously expressed, including astrocytes [76, 77], but BACE1 expression in the brain is largely restricted to neurons [73]. Thus, if BACE1 cleavage – and not alternative splicing – is indeed a major mechanism of sgp130 production in the brain, sgp130 should not be released from primary astrocytes. Indeed, while astrocytes showed even higher levels of the full-length, membrane-bound form of gp130 compared to neurons, they did not secrete sgp130, as shown by immunoblot and ELISA (Fig. 4c-f) and did not respond to C3 treatment with an increase of full-length gp130 in the lysate. Taken together, BACE1 controls abundance of full-length gp130 in neurons and its shedding as sgp130.

### BACE1 cleavage attenuates neuronal gp130 signaling

**Fig. 5:**
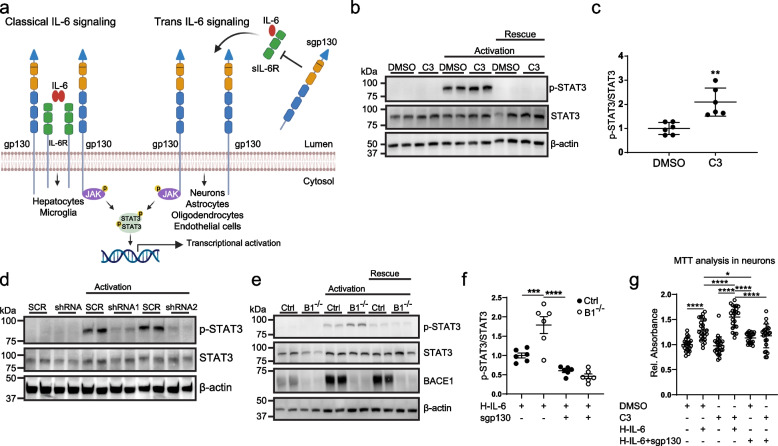
IL6 trans signaling in neurons is affected by BACE1 inhibition. **a** Classical and trans-signaling of IL-6. In classical signaling, IL-6 binds to the transmembrane IL-6R. The IL-6/IL-6R complex associates with the signal transducing protein gp130, initiating intracellular signaling involving the phosphorylation of STAT3 and downstream transcription. In trans-signaling, a soluble form of IL-6R (sIL-6R) is proteolytically released by the metalloprotease ADAM10/17 or by alternative splicing from specific cells (such as microglia in the brain). sIL-6R binds IL-6 to form a complex that signals through gp130. The BACE1-cleaved sgp130 ectodomain can also bind to the IL-6/IL-6R complex and specifically inhibit trans-signaling without affecting classic IL-6 signaling. **b** immunoblots for phosphorylated STAT3 (p-STAT3) and STAT3 in neuronal lysates following activation of trans-signaling with 10 ng/ml of the IL-6/IL6R complex fusion protein hyper IL-6 (H-IL-6) for 15 min, in the presence or absence of BACE inhibitor C3. For the rescue experiment, H-IL-6 was preincubated with sgp130 before the addition. Actin serves as loading control. **c** Quantification of p-STAT3/total STAT3 ratio in samples from **b** (*N* = 6 biological replicates). Shown are mean ± SD. Unpaired Student t-test. **d** Immunoblots against the indicated proteins in neurons transduced with lentiviruses expressing scrambled (SCR) control shRNA or two distinct shRNAs against gp130 (shRNA1, shRNA2). Activation of gp130 signaling with H-IL-6 was done as in **b**. **e** Immunoblots against indicated proteins in floxed BACE1 (Ctrl) or BACE1^−/−^ neurons (lentivirally Cre-transduced). Activation of gp130 signaling with H-IL-6 and rescue with sg130 was done as in **b**. **f** Quantification of p-STAT3/total STAT3 ratio in samples from **e** (*N* = 6 biological replicates). Shown are mean ± SD. One-way ANOVA with post hoc Tukey’s multiple comparison test. **g** MTT measurement of primary neurons at DIV12 after culture in the absence of serum-like B27 and in the absence or presence of BACE inhibitor C3 upon activation with H-IL-6 or rescue with sgp130. Shown are mean ± SD from *N* = 24 biological replicates. One-way ANOVA with post hoc Tukey’s multiple comparison test. *: *p* < 0.05; ***: *p* < 0.001; ****: *p* < 0.0001

Because we found that BACE1 controls neuronal abundance of gp130, we tested whether BACE1 affects neuronal gp130 signaling. gp130 is a cell surface cytokine receptor and has a dual role in IL-6 signaling [71, 78, 79]. In the classical signaling mode, the soluble cytokine IL-6 binds its direct receptor, the membrane protein IL-6R (Fig. 5a). The IL-6/IL-6R complex then associates with gp130 in the same cell, which dimerizes and activates signaling, in particular through JAK1/STAT3. While gp130 is expressed in nearly all cell types, most of them, including neurons, do not express IL-6R and, thus, cannot signal in the classical mode. However, neurons can still be activated by a soluble IL-6/IL-6R complex (trans signaling mode, Fig. 5a), because IL-6R can be shed from other cell types by the metalloprotease ADAM17 [80] and be present as soluble IL-6R. The shed sIL-6R can bind the ligand IL-6. Together, the complex of IL-6 and sIL-6R can associate with cellular gp130 and subsequently induce gp130 dimerization and signaling. Interestingly, IL-6 trans-signaling, but not IL-6 classic-signaling can be inhibited by sgp130 [79].

To test for a role of BACE1-mediated cleavage in gp130 signaling, we used primary murine neurons and activated them with the commonly used hyperIL-6 (H-IL-6), a fusion protein of IL-6 and soluble IL-6R, which can activate cells in the absence of membrane-bound IL-6R [81]. H-IL-6 activated JAK-STAT signaling as seen with strongly increased p-STAT3 levels, which were further enhanced by about two-fold upon addition of the BACE1 inhibitor C3 (Fig. 5b and c). This activation was prevented by addition of a recombinant sgp130 fusion protein, which sequesters H-IL-6 [79], or by shRNA-mediated knock-down of gp130 (Fig. 5d), demonstrating that activation of the JAK-STAT pathway was mediated through gp130 and not other BACE1 substrates. Increased activation by H-IL-6 was also obtained when BACE1 was genetically blocked using floxed BACE1 KO neurons transduced with a Cre recombinase-expressing lentivirus (Fig. 5e, f).

Gp130 signaling is neuroprotective, for example under excitotoxic or growth factor-withdrawal conditions [82–84]. To investigate whether this function is also modulated by BACE1 inhibition, we cultured primary neurons without the addition of the serum-like supplement B27 and scored neuronal survival with an MTT assay, where a higher signal corresponds to more cell survival. Addition of H-IL-6 increased the MTT signal and this was further enhanced upon BACE1 inhibition (Fig. 5g and Suppl. Fig. S5), demonstrating that BACE1 inhibition increases neuronal survival through gp130 signaling.

Taken together, a loss of BACE1 activity enhances gp130 signaling, which is in line with the increased abundance of gp130 and the concomitant reduction of shed sgp130.

## Discussion

This study establishes the cytokine receptor gp130 as an in vivo substrate of the protease BACE1 in animals and human CNS and demonstrates a novel biological concept, namely that BACE1 cleavage acts as a mechanism to attenuate gp130 function and IL-6 signaling. Additionally, our study has major implications for the development of BACE1-targeted therapies in Alzheimer’s disease. The measurement of the soluble, BACE1-cleaved forms of gp130 and SEZ6, in addition to APP products, provides a more complete fingerprint of BACE1 activity towards its major substrates, and suggests the use of sgp130 and sSEZ6 as rapidly responding pharmacodynamic activity markers for BACE1 in vivo.

The cytokine receptor gp130 exists as a transmembrane protein mediating signaling and as a soluble form comprising most of its ectodomain. The soluble form is found in blood and in the conditioned medium of cultured cells [70, 85] and was thought to result predominantly from alternative splicing [71, 86]. Our study reveals that instead more than half of the sgp130 in CSF, plasma and in the conditioned media of primary neurons is in fact generated through BACE1-mediated proteolytic shedding of the transmembrane form of gp130. This establishes a new mode of sgp130 generation in vitro and in vivo. The remaining sgp130 after BACE1 inhibition or knock-out may result from alternative splicing or potentially through proteolytic cleavage by as yet unidentified proteases. Indeed, in human immortalized hepatocytes, ADAM proteases appear to contribute to a minor extent to sgp130 release [71]. This is similar to other membrane proteins, such as APP, that are mainly shed by one protease, but may additionally be shed by other proteases, often to a smaller extent [26]. However, sgp130 was not significantly changed in the serum of hypomorphic ADAM17 knock-out mice [71] or in the conditioned medium of neurons lacking ADAM10 or active ADAM17 [65, 87] or in CSF of mice lacking active ADAM17 [87]. Generation of antibodies specifically binding the C-terminus of the spliced or the BACE1-cleaved sgp130 may help to set up assays that distinguish the proteolytically generated and the differentially spliced sgp130 forms.

For many substrates of BACE1 and other shedding proteases, it remains unclear whether their function is altered as a result of their proteolytic cleavage [26]. In contrast, for neuronal gp130 we now demonstrate that BACE1 attenuates gp130 signaling as evidenced by increased JAK/STAT signaling and enhanced neuronal survival upon BACE1 deletion or inhibition. Mechanistically, BACE1 cleavage may reduce gp130 signaling in two ways, a) by lowering the abundance of the full-length receptor gp130 and b) by generating sgp130, which can act as a decoy receptor as seen in our rescue experiments, but also demonstrated in vivo, where a Fc fusion protein of sgp130 is used to therapeutically lower IL6 trans signaling in mouse models of inflammatory diseases and cancer and in clinical trials for inflammatory bowel disease [88, 89].

Signaling of gp130 transmits signals of the IL-6 family of cytokines and has essential homeostatic and protective roles, for example in inflammation, metabolism or neural development [42, 43, 90, 91]. The new role of BACE1 as a regulator of gp130 signaling raises the possibility that BACE1 contributes to known functions of gp130 signaling, in particular in the nervous system, where BACE1 is highly expressed primarily in neurons. Most of the gp130 signaling functions in neurons were studied in in vitro systems, but a few studies used rodents which allow a phenotypic comparison to BACE1-deficient mice. One example is the gp130 signaling-dependent generation of astrocytes from stem cells in the mouse brain [83]. Thus, we predict that BACE1-deficiency, which reduces sgp130 levels resulting in increased IL6 trans-signaling, may similarly lead to increased astrogenesis in mice, which was indeed observed [92]. Although increased Notch signaling due to reduced cleavage of the BACE1 substrate Jagged1 was proposed as an underlying mechanism, an additional contribution of gp130 signaling appears possible given the multiple and complex control mechanisms of astrocyte induction [90].

IL-6 can also reduce body weight through stimulation of gp130 trans signaling in the hypothalamus [93–97]. A reduced body weight is also seen for mice deficient in BACE1 and for participants of clinical trials treated chronically with a BACE1-targeted inhibitor [59, 98–100]. This raises the intriguing possibility that BACE1-controlled gp130 signaling specifically in hypothalamic neurons, where BACE1 is also expressed, regulates metabolism and controls body weight in addition to distinct BACE1 substrates, such as the insulin receptor, or BACE1 cleavage products, such as Aβ, which have also been implicated in this process [100–103]. Importantly, the majority of IL-6 signaling in the brain depends on IL-6 trans-signaling [104].

Besides a reduced body weight, several BACE1-targeted inhibitors tested in advanced clinical trials induced a mild, non-progressive and reversible cognitive worsening, which is seen as an unacceptable side effect that needs to be understood and defined by predictive biomarkers before BACE inhibitors may be tested in future prevention trials for AD [9]. BACE inhibitors also mildly enhanced other adverse events, such as psychiatric symptoms. Whether reduced BACE1 cleavage of gp130 or enhanced g130 signaling contribute to the adverse events is unknown. A clear proof is difficult or even impossible, given that cleavage products of several other BACE1 substrates, including SEZ6, CHL1, APP and NRG3 also have established synaptic functions [105], and thus, may additionally contribute to the adverse events resulting from BACE1 inhibition. In the murine nervous system, IL-6 trans signaling, which acts through gp130, can have detrimental effects, such as increased blood brain barrier leakage, gliosis, impaired hippocampal neurogenesis and cerebellar neurodegeneration [104, 106], but also neuroprotective effects as shown in our study and in previous ones [82–84]. It is conceivable that chronic BACE inhibition, which lowers sgp130 release, sensitizes treated individuals to the detrimental, but also beneficial effects of IL-6 signaling, given that sgp130 forms a buffer system preventing IL-6 trans signaling-mediated gp130 activation under healthy conditions, where IL-6 concentration is low [88]. This buffering is overcome when IL-6 concentration strongly increases during infection or inflammation, and reaches – together with sIL-6R – molar concentrations similar to sgp130. Upon BACE1 inhibition, sgp130 shedding is strongly reduced and likely attenuates the IL-6 buffering system. Whether a resulting enhanced IL-6 sensitivity upon BACE inhibition contributes to the adverse events of BACE inhibitors remains unknown, but increased, pro-inflammatory IL-6 signaling is linked to cognitive decline in AD [107]. One way to address a possible involvement of sgp130 is to measure sgp130 in CSF or even in plasma from the Phase 3 clinical trials, where cognitive worsening was observed and to correlate the extent of its lowering to the occurrence of side effects. In case of a positive correlation, sgp130 and other CSF biomarkers of BACE1 inhibition may be used as a pharmacodynamic BACE1 activity marker to individually adjust the doses in a precision medicine approach with the aim of reducing side effects. In fact, preclinical studies suggest that 50% or less inhibition of BACE1 may be tolerable and still be able to reduce accrual of amyloid plaques [9].

Besides neuronal IL-6 signaling, BACE1 may also control IL-6 signaling in tumor-promoting macrophages in glioblastoma [7]. Inhibition of BACE1 suppressed glioblastoma growth by stimulating macrophage phagocytosis of glioblastoma stem cells. This phenotype required BACE1 cleavage of IL-6R, which is expressed in macrophages, but not neurons [76], making BACE1 a potential drug target for glioblastoma. Whether BACE1 cleavage of gp130 also contributes to IL-6 signaling in macrophages, was not investigated. This recent study and our work demonstrate that BACE1 controls IL-6 signaling and that the molecular mechanisms differ in a cell type-dependent manner. Moreover, it will be interesting whether the lowering of sgp130 levels in the blood after BACE1 inhibition will have a consequence on the susceptibility to IL-6 signaling in inflammatory and infectious states [108].

## Conclusion

Our pharmacoproteomics study has important implications for translational research beyond BACE1 and gp130. In preclinical and clinical drug development, it is essential to measure in vivo drug treatment responses, referred to as pharmacodynamics (PD). This includes monitoring of target engagement of the disease-relevant drug target, but also of on-target adverse or even beneficial events due to the biology of the drug target which are difficult to predict. A previous example is the protease γ-secretase where development of inhibitors was discontinued as AD treatment because they did not only block APP cleavage, but also cleavage and function of other γ-secretase substrates, in particular Notch [109, 110]. In contrast to commonly used counter-screens, pharmacoproteomics is not restricted to individual proteins that need to be known previously for selected monitoring, but instead can identify and quantify such proteins (e.g. substrates) in an unbiased manner and critically test many proteins simultaneously, even in a time- and dose-dependent manner, as shown here for the protease BACE1 with diverse substrates such as gp130, SEZ6, and VCAM1. Additionally, pharmacoproteomics allows to identify potential off-target effects in a proteome-wide manner, as seen with the increased HBA and HBB levels upon dosing of MBI-4, but not verubecestat, in the NHP CSF. Given the significant cost of drug development and the potential failure of drugs at different steps in the development process, we suggest the routine incorporation of NHP CSF pharmacoproteomics into preclinical neuroscience drug development.

## Supplementary Information

**Additional file 1: Suppl. Tab. 1.** Identified transmembrane type 1 proteins. (UniProt subcellular location: Single-pass type I membrane protein [SL-9905], *p*-values < 0.05 are displayed red). **Suppl. Tab. 2.** Identified transmembrane type 2 proteins. (UniProt subcellular location: Single-pass type II membrane protein [SL-9906], *p*-values < 0.05 are displayed red). **Suppl. Tab. 3.** Identified multi-pass transmembrane proteins. (UniProt subcellular location: Single-pass type I membrane protein [SL-9909], *p*-values < 0.05 are displayed red, * transmembrane regions not annotated at UniPro.

**Additional file 2: Supplementary Data 1.** CSF proteomics of NHP treated with the BACE inhibitor MBI-4.

**Additional file 3: Supplementary Data 2.** CSF proteomics of NHP treated with the BACE inhibitor verubecestat.

**Additional file 4: Supplementary Fig. S1.** CSF proteomics of MBI-4 or vehicle-treated NHP.

**Additional file 5: Supplementary Fig. S2.** Extended plots for NHP CSF proteomics in response to MBI-4 and verubecestat.

**Additional file 6: Supplementary Fig. S3.** Pharmacokinetics and -dynamics of NHP treated with verubecestat.

**Additional file 7: Supplementary Fig. S4.** Full Western blots of sSEZ6 in human CSF after BACE inhibition.

**Additional file 8: Supplementary Fig. S5.** Increased survival of neurons can be blocked by sgp130.

## Data Availability

The mass spectrometry proteomics data of the MBI-4 study have been deposited to the ProteomeXchange Consortium via the PRIDE [111] partner repository with the dataset identifier PXD035141.

## Abbreviations

Aβ: Amyloid beta peptide
AD: Alzheimer’s disease
ADAM10: Disintegrin and metalloproteinase domain-containing protein 10
ADAM17: Disintegrin and metalloproteinase domain-containing protein 17
AGC: Automatic gain control
APLP1: Amyloid beta precursor like protein 1
APLP2: Amyloid beta precursor like protein 2
APP: Amyloid precursor protein
BACE1: β-site APP cleaving enzyme 1
BACE2: β-site APP cleaving enzyme 2
BCA: Bicinchoninic acid assay
BSG: Basigin
CACHD1: VWFA and cache domain-containing protein 1
CADM4: Cell adhesion molecule 4
CD5: Cluster of differentiation 5, T-cell surface glycoprotein CD5
CNS: Central nervous system
CNTN2: Contactin-2
Cre: Recombinase cre
CSF: Cerebrospinal fluid
CSF1R: Macrophage colony-stimulating factor 1 receptor
CHL1: Neural cell adhesion molecule L1-like protein
DBCO: Dibenzocyclooctyne
DIV: Days in-vitro
DMSO: Dimethylsulfoxid
E15/E16: Embryo at day 15/16
EDTA: Ethylenediaminetetraacetic acid
ELISA: Enzyme-linked Immunosorbent Assay
FASP: Filter assisted sample preparation
FDR: False discovery rate
FGFR1: Fibroblast growth factor receptor 1
gp130: Glycoprotein 130
H-IL-6: Hyper interleukin-6, IL-6/IL6R complex fusion protein
HBA: Hemoglobin subunit alpha
HBB: Hemoglobin subunit beta
IL-6: Interleukin-6
IL6-R: Interleukin-6 receptor subunit alpha
IL6ST: Interleukin-6 receptor subunit beta
JAK: Janus kinase
L1CAM: Neural cell adhesion molecule L1
LC-MS/MS: Liquid chromatography tandem mass spectrometry
LFQ: Label-free quantification
LRRN1: Leucine-rich repeat neuronal protein 1
mRNA: Messenger ribonucleic acid
MS: Mass spectrometry
MTT: 3-(4,5-dimethylthiazol-2-yl)-2,5-diphenyltetrazolium bromide
NHP: Non-human primate
NRCAM: Neuronal cell adhesion molecule
PAGE: Polyacrylamide gel electrophoresis
PTPRG: Receptor-type tyrosine-protein phosphatase gamma
PVDF: Polyvinylidenfluorid
QARIP: Quantitative Analysis of Regulated Intramembrane Proteolysis
RIPA: Radioimmunoprecipitation assay
sAPPα: Soluble amyloid precursor protein α
sAPPβ: Soluble amyloid precursor protein β
SDS: Sodium dodecylsulfate
SEZ6: Seizure protein 6 homolog
SEZ6L: Seizure 6-like protein
shRNA: Short hairpin ribonucleic acid
SPECS: Secretome protein enrichment with click sugars
STAT3: Signal transducer and activator of transcription 3
TBS: Tris buffered saline
TGFBR2: TGF-beta receptor type-2
TGOLN2: Trans-Golgi network integral membrane protein 2
TMT: Tandem mass tags
VCAM1: Vascular cell adhesion protein 1
