# Supplementary material for: The Alzheimer’s disease-linked protease BACE1 modulates neuronal IL-6 signaling through shedding of the receptor gp130
Source: Mol Neurodegener. 2023 Feb 21;18:13. doi: 10.1186/s13024-023-00596-6 (PMC9942414; doi:10.1186/s13024-023-00596-6)
Supplement: Supplementary file 1 — Additional file 1: Suppl. Tab. 1. Identified transmembrane type 1 proteins. (UniProt subcellular location: Single-pass type I membrane protein [SL-9905], p-values < 0.05 are displayed red). Suppl. Tab. 2. Identified transmembrane type 2 proteins. (UniProt subcellular location: Single-pass type II membrane protein [SL-9906], p-values < 0.05 are displayed red). Suppl. Tab. 3. Identified multi-pass transmembrane proteins. (UniProt subcellular location: Single-pass type I membrane protein [SL-9909], p-values < 0.05 are displayed red, * transmembrane regions not annotated at UniPro. [file 13024_2023_596_MOESM1_ESM.pdf]

Supplementary Tables

Index

Suppl. Tab. 1: Identified transmembrane type 1 proteins. (UniProt subcellular location: Single-pass type I membrane protein [SL-9905], p-values < 0.05 are displayed red) 2
Suppl. Tab. 2: Identified transmembrane type 2 proteins. (UniProt subcellular location: Single-pass type II membrane protein [SL-9906], p-values < 0.05 are displayed red) 9
Suppl. Tab. 3: Identified multi-pass transmembrane proteins. (UniProt subcellular location: Single-pass type I membrane protein [SL-9909], p-values < 0.05 are displayed red, * transmembrane regions not annotated at UniProt) 11

Color code for schematic sequence in in the column Topology (derived by QARIP http://webclu.bio.wzw.tum.de/qarip/):

Signalpeptide

Extracellular domain

Transmembrane domain

Cytoplasmic domain

Lumenal domain

Propeptide

Unknown

detected peptides

Suppl. Tab. 1: Identified transmembrane type 1 proteins. (UniProt subcellular location: Single-pass type I membrane protein [SL-9905], p-values < 0.05 are displayed red)

UniProt AC (*M. mulatta*)	UniProt AC (*H. sapiens*)	Protein names (human homologs)	Gene names (human homologs)	Unique peptides	Ratio (100 mpk/Veh @ 24 h)	p-value (100 mpk/Veh @ 24 h)	Topology (Phobius, corrected according to UniProt topology for human homologs)
F7F6J3	Q53EL9	Seizure protein 6 homolog	SEZ6	10	0.36	3.64E-06	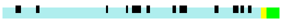
F7FXB6	P40189	Interleukin-6 receptor subunit beta	IL6ST	15	0.46	1.45E-03	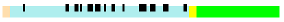
F6W7X9	P19320	Vascular cell adhesion protein 1	VCAM1	21	0.51	4.20E-03	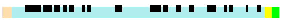
F7GPP8	Q9BYH1	Seizure 6-like protein	SEZ6L	13	0.68	1.87E-03	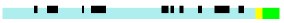
A0A5F7ZLI9	P32004	Neural cell adhesion molecule L1	L1CAM	8	0.69	1.35E-01	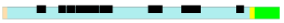
F6R6D9	Q6UXK5	Leucine-rich repeat neuronal protein 1	LRRN1	6	0.69	6.12E-02	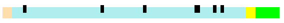

UniProt AC (*M. mulatta*)	UniProt AC (*H. sapiens*)	Protein names (human homologs)	Gene names (human homologs)	Unique peptides	Ratio (100 mpk/Veh @ 24 h)	p-value (100 mpk/Veh @ 24 h)	Topology (Phobius, corrected according to UniProt topology for human homologs)
F7A4T4	P11362	Fibroblast growth factor receptor 1	FGFR1	7	0.71	2.40E-03	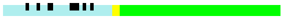
A0A5F7Z806	P10721	Mast/stem cell growth factor receptor Kit	KIT	6	0.71	4.38E-02	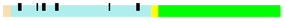
F7FJ90	Q06481	Amyloid-like protein 2	APLP2	24	0.72	7.79E-03	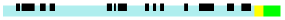
F7G9L5	O60462	Neuropilin-2	NRP2	14	0.75	1.80E-01	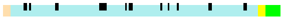
F7ELT5	P05067	Amyloid beta A4 protein	APP	26	0.75	1.85E-03	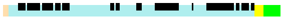
A0A1D5QZV9	P51693	Amyloid-like protein 1	APLP1	33	0.76	1.20E-02	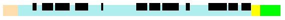
F7HHG1	P52799	Ephrin-B2	EFNB2	5	0.77	9.72E-02	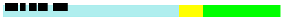
F7H285	P10586	Receptor-type tyrosine-protein phosphatase F	PTPRF	8	0.77	3.67E-02	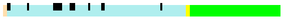
P60030	O43493	Trans-Golgi network integral membrane protein 2	TGOLN2	10	0.78	3.72E-01	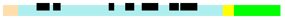
F7GI13	Q8IW52	SLIT and NTRK-like protein 4	SLITRK4	7	0.79	1.01E-01	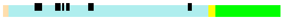
F6ZB61	Q96KG7	Multiple epidermal growth factor-like domains protein 10	MEGF10	18	0.80	6.65E-02	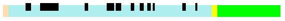
F7GXZ0	Q16653	Myelin-oligodendrocyte glycoprotein	MOG	3	0.80	9.95E-02	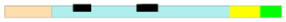
F7DWH2	P23468	Receptor-type tyrosine-protein phosphatase delta	PTPRD	16	0.81	4.02E-03	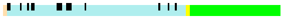
A0A1D5QGI4	Q6UXD5	Seizure 6-like protein 2	SEZ6L2	10	0.81	7.17E-02	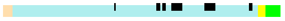
F6UXR7	Q8NFT8	Delta and Notch-like epidermal growth factor-related receptor	DNER	6	0.82	1.28E-01	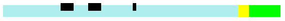
F7EHH7	P23470	Receptor-type tyrosine-protein phosphatase gamma	PTPRG	9	0.83	4.15E-03	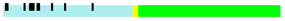
F7B8S4	O00533	Neural cell adhesion molecule L1-like protein	CHL1	38	0.83	1.37E-02	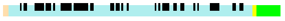
F6UWH5	Q92932	Receptor-type tyrosine-protein phosphatase N2	PTPRN2	9	0.84	3.34E-02	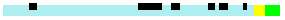
F6S8N3	Q96PX8	SLIT and NTRK-like protein 1	SLITRK1	7	0.84	3.18E-01	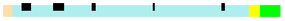
F7F5S9	Q9NY47	Voltage-dependent calcium channel subunit alpha-2/delta-2	CACNA2D2	10	0.84	1.72E-01	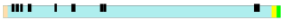
F6ZPW9	Q8N3J6	Cell adhesion molecule 2	CADM2	9	0.85	1.76E-01	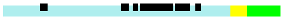

UniProt AC (*M. mulatta*)	UniProt AC (*H. sapiens*)	Protein names (human homologs)	Gene names (human homologs)	Unique peptides	Ratio (100 mpk/Veh @ 24 h)	p-value (100 mpk/Veh @ 24 h)	Topology (Phobius, corrected according to UniProt topology for human homologs)
F7DB43	Q14574	Desmocollin-3	DSC3	4	0.85	3.59E-01	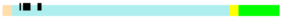
F7E1W0	Q08174	Protocadherin-1	PCDH1	10	0.86	6.89E-02	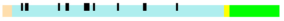
A0A1D5QL11	P54764	Ephrin type-A receptor 4	EPHA4	15	0.86	6.80E-02	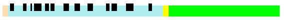
F6RM33	P13591	Neural cell adhesion molecule 1	NCAM1	5	0.86	1.81E-01	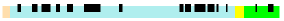
F6TPB6	Q9UMF0	Intercellular adhesion molecule 5	ICAM5	5	0.86	2.59E-01	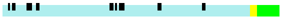
A0A1D5R7E9	Q9H8J5	MANSC domain-containing protein 1	MANSC1	5	0.87	4.48E-01	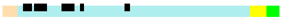
F6TXL3	Q8TCZ2	CD99 antigen-like protein 2	CD99L2	4	0.87	1.98E-01	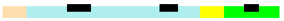
A0A1D5QFH7	P21802	Fibroblast growth factor receptor 2	FGFR2	7	0.87	1.84E-01	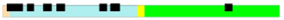
F7BTH1	P09619	Platelet-derived growth factor receptor beta	PDGFRB	7	0.87	1.69E-01	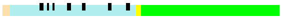
A0A5K1UBA0	O60939	Sodium channel subunit beta-2	SCN2B	2	0.88	4.66E-01	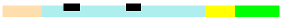
F7H047	Q16849	Receptor-type tyrosine-protein phosphatase-like N	PTPRN	9	0.88	3.10E-01	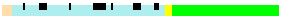
A0A5F7ZDV7	P78324	Tyrosine-protein phosphatase non-receptor type substrate 1	SIRPA	6	0.88	1.61E-02	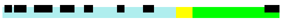
F7E9U2	Q13740	CD166 antigen	ALCAM	17	0.89	8.11E-02	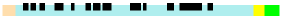
F6Z7U0	Q9P2S2	Neurexin-2	NRXN2	19	0.89	7.08E-02	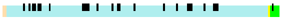
F6Q7Q5	Q8NFZ8	Cell adhesion molecule 4	CADM4	8	0.89	4.72E-02	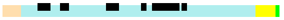
F7HMX4	O75509	Tumor necrosis factor receptor superfamily member 21	TNFRSF21	4	0.90	4.46E-01	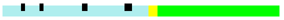
F7DNG4	Q9BQT9	Calsyntenin-3	CLSTN3	12	0.90	2.35E-01	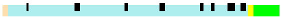
F6VQT9	Q16620	BDNF/NT-3 growth factors receptor	NTRK2	5	0.90	5.94E-01	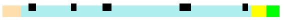
A0A5F8AGU6	Q8N126	Cell adhesion molecule 3	CADM3	10	0.90	1.66E-01	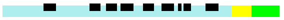
F7AVS7	Q9Y5Y7	Lymphatic vessel endothelial hyaluronic acid receptor 1	LYVE1	5	0.91	2.27E-01	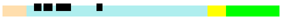
A0A1D5RGN2	Q9BY67	Cell adhesion molecule 1	CADM1	7	0.91	2.80E-01	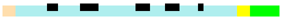

UniProt AC (*M. mulatta*)	UniProt AC (*H. sapiens*)	Protein names (human homologs)	Gene names (human homologs)	Unique peptides	Ratio (100 mpk/Veh @ 24 h)	p-value (100 mpk/Veh @ 24 h)	Topology (Phobius, corrected according to UniProt topology for human homologs)
F6TNL5	P07333	Macrophage colony-stimulating factor 1 receptor	CSF1R	13	0.91	1.08E-01	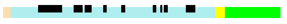
F6WAA5	P20916	Myelin-associated glycoprotein	MAG	7	0.91	2.56E-01	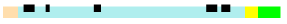
F7GLX3	Q03167	Transforming growth factor beta receptor type 3	TGFBR3	8	0.91	3.47E-01	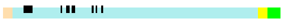
F7ADF9	Q92859	Neogenin	NEO1	25	0.91	1.70E-01	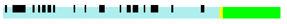
F6RU72	Q14118	Dystroglycan	DAG1	15	0.92	1.65E-01	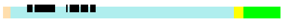
A0A1D5QF92	Q07954	Prolow-density lipoprotein receptor-related protein 1	LRP1	50	0.92	2.76E-01	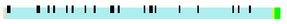
F6V7H4	Q92823	Neuronal cell adhesion molecule	NRCAM	44	0.93	1.26E-01	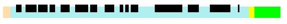
F7HR49	Q9HDB5	Neurexin-3-beta	NRXN3	3	0.93	8.62E-01	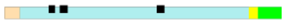
F7C6B8	Q9Y6N7	Roundabout homolog 1	ROBO1	10	0.93	5.67E-01	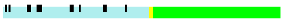
F6WI86	P19022	Cadherin-2	CDH2	16	0.93	4.34E-01	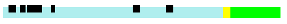
F7HP57	Q9NPR2	Semaphorin-4B	SEMA4B	17	0.94	4.12E-01	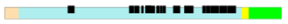
F7HBK3	Q15223	Nectin-1	PVRL1	5	0.94	3.86E-01	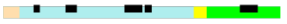
F6THA2	Q96AP7	Endothelial cell-selective adhesion molecule	ESAM	5	0.95	6.40E-01	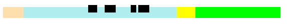
F7FQ55	Q12907	Vesicular integral-membrane protein VIP36	LMAN2	12	0.95	3.20E-01	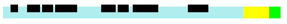
F7HGA1	Q13332	Receptor-type tyrosine-protein phosphatase S	PTPRS	15	0.95	6.71E-01	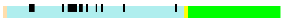
P55245	P00533	Epidermal growth factor receptor	EGFR	6	0.95	8.03E-01	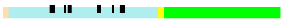
F6RRI0	P43146	Netrin receptor DCC	DCC	13	0.95	3.12E-01	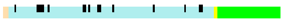
F7AGQ0	Q9H2A7	C-X-C motif chemokine 16	CXCL16	5	0.95	7.19E-01	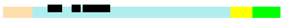
F6ZDX2	P16070	CD44 antigen	CD44	4	0.95	1.98E-01	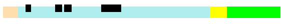
F6SYF6	Q9Y4C0	Neurexin-3-alpha	NRXN3	23	0.96	5.58E-01	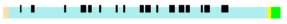
F6RSK7	Q6UXB8	Peptidase inhibitor 16	PI16	8	0.96	5.96E-01	
F7HKZ9	P09603	Macrophage colony-stimulating factor 1	CSF1	9	0.97	4.45E-01	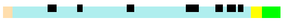

UniProt AC (*M. mulatta*)	UniProt AC (*H. sapiens*)	Protein names (human homologs)	Gene names (human homologs)	Unique peptides	Ratio (100 mpk/Veh @ 24 h)	p-value (100 mpk/Veh @ 24 h)	Topology (Phobius, corrected according to UniProt topology for human homologs)
F7HIJ2	Q9Y279	V-set and immunoglobulin domain-containing protein 4	VSIG4	7	0.97	6.14E-01	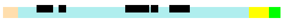
F7A6D9	Q8WY21	VPS10 domain-containing receptor SorCS1	SORCS1	13	0.97	8.05E-01	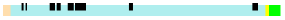
F6PK24	P22897	Macrophage mannose receptor 1	MRC1	24	0.98	7.90E-01	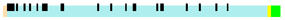
F7E8S5	O15031	Plexin-B2	PLXNB2	20	0.98	2.65E-01	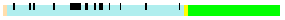
F7DAU3	Q86VB7	Scavenger receptor cysteine-rich type 1 protein M130	CD163	27	0.98	8.48E-01	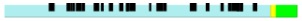
H9H377	A6NLU5	V-set and transmembrane domain-containing protein 2B	VSTM2B	4	0.98	8.34E-01	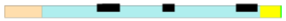
F6SH47	Q6UX71	Plexin domain-containing protein 2	PLXDC2	16	0.98	8.90E-01	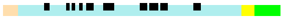
F7HLV3	Q9NZ53	Podocalyxin-like protein 2	PODXL2	3	0.98	8.65E-01	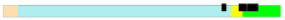
F7FZR9	P22607	Fibroblast growth factor receptor 3	FGFR3	7	0.99	8.70E-01	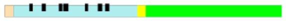
F7GY03	Q8NFY4	Semaphorin-6D	SEMA6D	6	0.99	9.19E-01	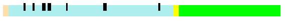
F7E6Z1	Q9NYQ8	Protocadherin Fat 2	FAT2	38	0.99	9.23E-01	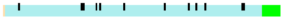
F7FHP2	Q9P2E7	Protocadherin-10	PCDH10	4	0.99	9.88E-01	
F7A8W3	P55286	Cadherin-8	CDH8	4	1.00	9.55E-01	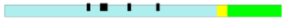
F7DJJ9	P23471	Receptor-type tyrosine-protein phosphatase zeta	PTPRZ1	19	1.00	9.45E-01	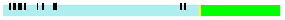
F7CEC9	Q8IUK5	Plexin domain-containing protein 1	PLXDC1	10	1.01	9.71E-01	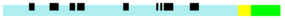
F7G7B5	Q24JP5	Transmembrane protein 132A	TMEM132A	23	1.01	9.29E-01	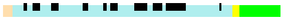
F7HEY4	O14917	Protocadherin-17	PCDH17	9	1.01	9.27E-01	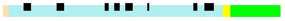
F6TNE5	Q7Z7M0	Multiple epidermal growth factor-like domains protein 8	MEGF8	48	1.01	8.60E-01	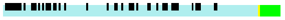
F6ZS69	P14209	CD99 antigen	CD99	5	1.01	8.03E-01	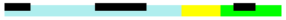
A0A1D5QQE5	O15394	Neural cell adhesion molecule 2	NCAM2	24	1.02	8.09E-01	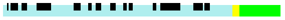
F7BDW8	P19021	Peptidyl-glycine alpha-amidating monooxygenase	PAM	33	1.02	4.04E-01	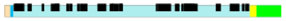

UniProt AC (*M. mulatta*)	UniProt AC (*H. sapiens*)	Protein names (human homologs)	Gene names (human homologs)	Unique peptides	Ratio (100 mpk/Veh @ 24 h)	p-value (100 mpk/Veh @ 24 h)	Topology (Phobius, corrected according to UniProt topology for human homologs)
F7C0W7	Q9H1U4	Multiple epidermal growth factor-like domains protein 9	MEGF9	6	1.02	8.70E-01	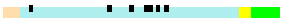
A0A1D5QVR4	P54289	Voltage-dependent calcium channel subunit alpha-2/delta-1	CACNA2D1	2	1.02	7.88E-01	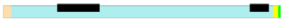
F7AR46	O60279	Sushi domain-containing protein 5	SUSD5	11	1.03	8.04E-01	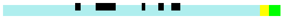
F7HB21	P55291	Cadherin-15	CDH15	13	1.03	6.35E-01	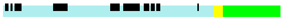
A0A1D5RL79	Q6EMK4	Vasorin	VASN	5	1.04	6.91E-01	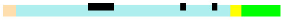
F6R206	Q9UK28	Transmembrane protein 59-like	TMEM59L	4	1.04	3.57E-01	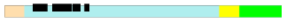
F7EMR9	O75787	Renin receptor	ATP6AP2	11	1.04	6.15E-01	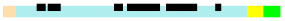
Q5NKV6	P05362	Intercellular adhesion molecule 1	ICAM1	4	1.04	4.32E-01	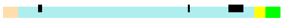
F7BCK9	P13473	Lysosome-associated membrane glycoprotein 2	LAMP2	4	1.05	4.92E-01	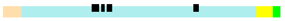
F7HC68	P08174	Complement decay-accelerating factor	CD55	10	1.06	5.74E-01	
F7DGM2	P55283	Cadherin-4	CDH4	5	1.06	4.88E-01	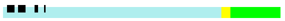
F7CNM1	Q96FE7	Phosphoinositide-3-kinase-interacting protein 1	PIK3IP1	5	1.06	5.08E-01	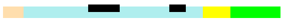
F7GRQ8	Q9Y5I4	Protocadherin alpha-C2	PCDHAC2	10	1.07	5.01E-01	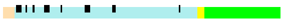
F6SM35	P43121	Cell surface glycoprotein MUC18	MCAM	17	1.07	6.43E-01	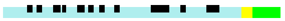
F7GFF0	P55287	Cadherin-11	CDH11	5	1.07	7.69E-01	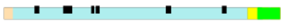
F6W3M2	P01871	Ig mu chain C region	IGHM	15	1.10	7.88E-01	
F6X837	O75882	Attractin	ATRN	14	1.11	3.63E-01	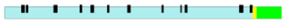
F6WDU0	Q9HC56	Protocadherin-9	PCDH9	8	1.12	5.35E-01	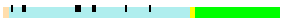
G7MUQ7	P55285	Cadherin-6	CDH6	7	1.20	4.67E-01	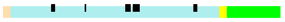
F6QFA6	Q9UPU3	VPS10 domain-containing receptor SorCS3	SORCS3	5	1.22	4.24E-03	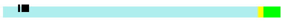
F6ZUX3	Q9C0A0	Contactin-associated protein-like 4	CNTNAP4	17	1.26	2.06E-01	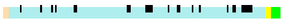
F7HN08	O60245	Protocadherin-7	PCDH7	5	1.30	4.06E-01	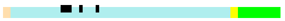

UniProt AC (*M. mulatta*)	UniProt AC (*H. sapiens*)	Protein names (human homologs)	Gene names (human homologs)	Unique peptides	Ratio (100 mpk/Veh @ 24 h)	p-value (100 mpk/Veh @ 24 h)	Topology (Phobius, corrected according to UniProt topology for human homologs)
F7E4X5	P30530	Tyrosine-protein kinase receptor UFO	AXL	3	1.36	2.36E-01	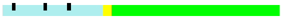
F6XNG5	Q9HCU0	Endosialin	CD248	6	1.46	3.58E-01	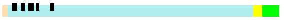
F7GSM9	Q9P0K1	Disintegrin and metalloproteinase domain-containing protein 22	ADAM22	3	1.48	4.45E-01	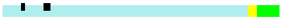
A0A1D5QFJ7	O14672	Disintegrin and metalloproteinase domain-containing protein 10	ADAM10	4	NaN	NaN	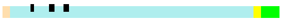
F6Y3S7	P35613	Basigin	BSG	5	NaN	NaN	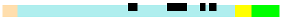
F6UWP6	Q86TY3	Uncharacterized protein C14orf37	C14orf37	2	NaN	NaN	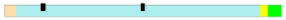
F7BKT3	O95196	Chondroitin sulfate proteoglycan 5	CSPG5	2	NaN	NaN	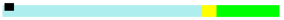
F7BC56	O75144	ICOS ligand	ICOSLG	2	NaN	NaN	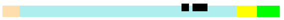
F6T7N0	Q12866	Tyrosine-protein kinase Mer	MERTK	4	NaN	NaN	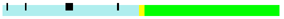
P60030	P58400	Neurexin-1-beta	NRXN1	4	NaN	NaN	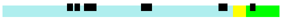
A0A1D5QMB8	O43157	Plexin-B1	PLXNB1	5	NaN	NaN	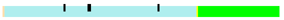
A0A1D5R0E5	Q92673	Sortilin-related receptor	SORL1	4	NaN	NaN	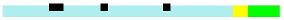
F6VLF7	Q99523	Sortilin	SORT1	6	NaN	NaN	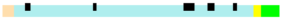

Suppl. Tab. 2: **Identified transmembrane type 2 proteins. (UniProt subcellular location: Single-pass type II membrane protein [SL-9906], p-values < 0.05 are displayed red)**

UniProt AC (*M. mulatta*)	UniProt AC (*H. sapiens*)	Protein names (human homologs)	Gene names (human homologs)	Unique peptides	Ratio (100 mpk/Veh @ 24 h)	p-value (100 mpk/Veh @ 24 h)	Topology (Phobius, corrected according to UniProt topology for human homologs)
F7GP66	Q16853	Membrane primary amine oxidase	AOC3	8	0.81	4.31E-01	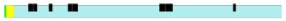
F6XJL0	Q8NBI6	Xyloside xylosyltransferase 1	XXYLT1	8	0.83	2.31E-01	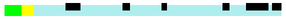
F7GY84	P05026	Sodium/potassium-transporting ATPase subunit beta-1	ATP1B1	3	0.84	9.86E-02	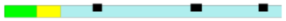
F6R9K4	Q00973	Beta-1,4 N-acetylgalactosaminyltransferase 1	B4GALNT1	5	0.87	2.56E-01	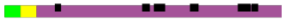
F7GN81	Q8NES3	Beta-1,3-N-acetylglucosaminyltransferase lunatic fringe	LFNG	12	0.88	2.53E-01	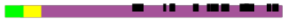
F6RUR9	Q5KU26	Collectin-12	COLEC12	5	0.88	4.44E-01	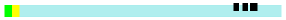
F6WPI5	P15291	Beta-1,4-galactosyltransferase 1	B4GALT1	7	0.88	4.20E-01	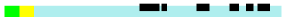
F7FZ79	Q9NX62	Inositol monophosphatase 3	IMPAD1	10	0.89	1.88E-01	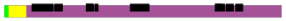
A0A1D5RIL8	P49641	Alpha-mannosidase 2x	MAN2A2	27	0.95	5.72E-01	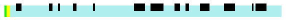
F7AT08	Q9NY97	UDP-GlcNAc:betaGal beta-1,3-N-acetylglucosaminyltransferase 2	B3GNT2	11	0.96	7.59E-01	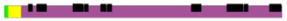
F6Q7P4	Q9Y644	Beta-1,3-N-acetylglucosaminyltransferase radical fringe	RFNG	5	0.96	7.12E-01	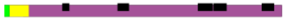
F7AP45	O95502	Neuronal pentraxin receptor	NPTXR	21	0.98	5.90E-01	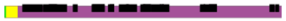
F6YY29	P33908	Mannosyl-oligosaccharide 1,2-alpha-mannosidase IA	MAN1A1	18	0.98	7.73E-01	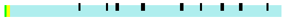
F6W932	P08195	4F2 cell-surface antigen heavy chain	SLC3A2	8	0.99	8.98E-01	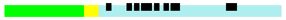
F7G642	O00468	Agrin	AGRN	17	1.00	9.92E-01	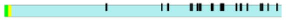
F7B3H7	Q10471	Polypeptide N-acetylgalactosaminyltransferase 2	GALNT2	11	1.00	9.91E-01	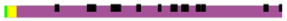
A0A5F8ACC7	Q8WVQ1	Soluble calcium-activated nucleotidase 1	CANT1	7	1.01	8.97E-01	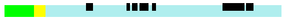
F6TT40	Q8IZP7	Heparan-sulfate 6-O-sulfotransferase 3	HS6ST3	8	1.01	9.41E-01	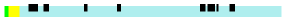
F6S245	Q9UBQ6	Exostosin-like 2	EXTL2	10	1.02	7.32E-01	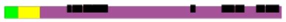

UniProt AC (*M. mulatta*)	UniProt AC (*H. sapiens*)	Protein names (human homologs)	Gene names (human homologs)	Unique peptides	Ratio (100 mpk/Veh @ 24 h)	p-value (100 mpk/Veh @ 24 h)	Topology (Phobius, corrected according to UniProt topology for human homologs)
F7EHM7	Q16706	Alpha-mannosidase 2	MAN2A1	10	1.05	7.28E-01	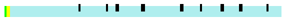
F6UX47	P02786	Transferrin receptor protein 1	TFRC	6	1.05	8.08E-01	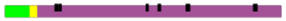
F6W2K4	Q8IV08	Phospholipase D3	PLD3	7	1.07	5.57E-01	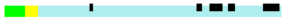
F7FX41	Q8NBJ4	Golgi membrane protein 1	GOLM1	13	1.08	3.72E-01	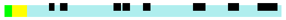
F6UCJ0	O75752	UDP-GalNAc:beta-1,3-N-acetylgalactosaminyltransferase 1	B3GALNT1	6	1.10	4.99E-01	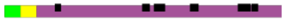
G7NC61	O43505	N-acetyllactosaminide beta-1,3-N-acetylglucosaminyltransferase	B3GNT1	21	1.11	2.49E-01	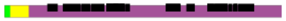
F7HGN6	P42658	Dipeptidyl aminopeptidase-like protein 6	DPP6	8	1.16	3.36E-01	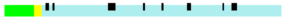
F7GR55	Q8WZA1	Protein O-linked-mannose beta-1,2-N-acetylglucosaminyltransferase 1	POMGNT1	15	1.16	2.44E-01	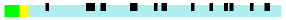
F7B0D0	Q9Y287	Integral membrane protein 2B	ITM2B	5	1.20	5.77E-01	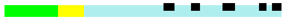
H9H468	O00461	Golgi integral membrane protein 4	GOLIM4	3	1.34	2.37E-01	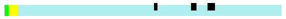
F7GGB1	Q96JF0	Beta-galactoside alpha-2,6-sialyltransferase 2	ST6GAL2	4	1.54	1.36E-01	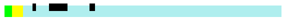
F6YR98	Q10472	Polypeptide N-acetylgalactosaminyltransferase 1	GALNT1	4	NaN	NaN	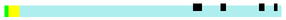
A0A1D5Q183	Q6P9A2	Polypeptide N-acetylgalactosaminyltransferase 18	GALNT18	6	NaN	NaN	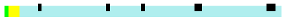
F6SE05	Q9NR34	Mannosyl-oligosaccharide 1,2-alpha-mannosidase IC	MAN1C1	2	NaN	NaN	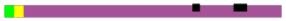
A0A1D5R5Y2	Q09328	Alpha-1,6-mannosylglycoprotein 6-beta-N-acetylglucosaminyltransferase A	MGAT5	4	NaN	NaN	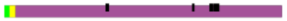

Suppl. Tab. 3: **Identified multi-pass transmembrane proteins. (UniProt subcellular location: Single-pass type I membrane protein [SL-9909], p-values < 0.05 are displayed red, * transmembrane regions not annotated at UniProt)**

UniProt AC (*M. mulatta*)	UniProt AC (*H. sapiens*)	Protein names (human homologs)	Gene names (human homologs)	Unique peptides	Ratio (100 mpk/Veh @ 24 h)	p-value (100 mpk/Veh @ 24 h)	Topology (Phobius, corrected according to UniProt topology for human homologs)
F7EBA3	Q9BZC7	ATP-binding cassette sub-family A member 2	ABCA2	4	0.73	1.17E-01	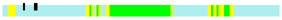
F6WDC7	Q9ULF5	Zinc transporter ZIP10	SLC39A10	7	0.75	1.86E-01	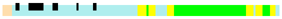
F7GXZ0	Q16653	Myelin-oligodendrocyte glycoprotein	MOG	3	0.80	9.95E-02	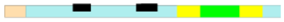
F6WUI7	Q8N6C5	Immunoglobulin superfamily member 1	IGSF1	3	0.84	1.18E-01	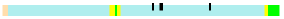
F7HQI7	Q9HAR2	Latrophilin-3	LPHN3	10	0.85	6.05E-02	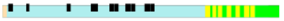
F7B4P4	O15354	Prosaposin receptor GPR37	GPR37	8	0.86	1.70E-01	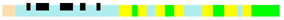
F7GRY2	P02748	Complement component C9	C9	25	0.99	9.19E-01	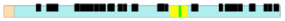
F6U5Q9	P07357	Complement component C8 alpha chain	C8A	14	1.00	9.72E-01	*
F7FAA5	O60241	Brain-specific angiogenesis inhibitor 2	BAI2	5	1.03	6.70E-01	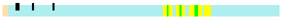
F7C0V2	O94910	Latrophilin-1	LPHN1	16	1.07	4.15E-01	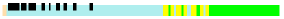
F7HJU3	Q5FWE3	Proline-rich transmembrane protein 3	PRRT3	6	1.16	7.43E-02	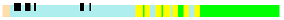
F6TEF3	O60883	Prosaposin receptor GPR37L1	GPR37L1	3	1.27	2.48E-01	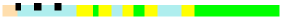
