# Supplementary material for: The Alzheimer’s disease-linked protease BACE1 modulates neuronal IL-6 signaling through shedding of the receptor gp130
Source: Mol Neurodegener. 2023 Feb 21;18:13. doi: 10.1186/s13024-023-00596-6 (PMC9942414; doi:10.1186/s13024-023-00596-6)
Supplement: Supplementary file 4 — Additional file 4: Supplementary Fig. S1. CSF proteomics of MBI-4 or vehicle-treated NHP. [file 13024_2023_596_MOESM4_ESM.pdf]

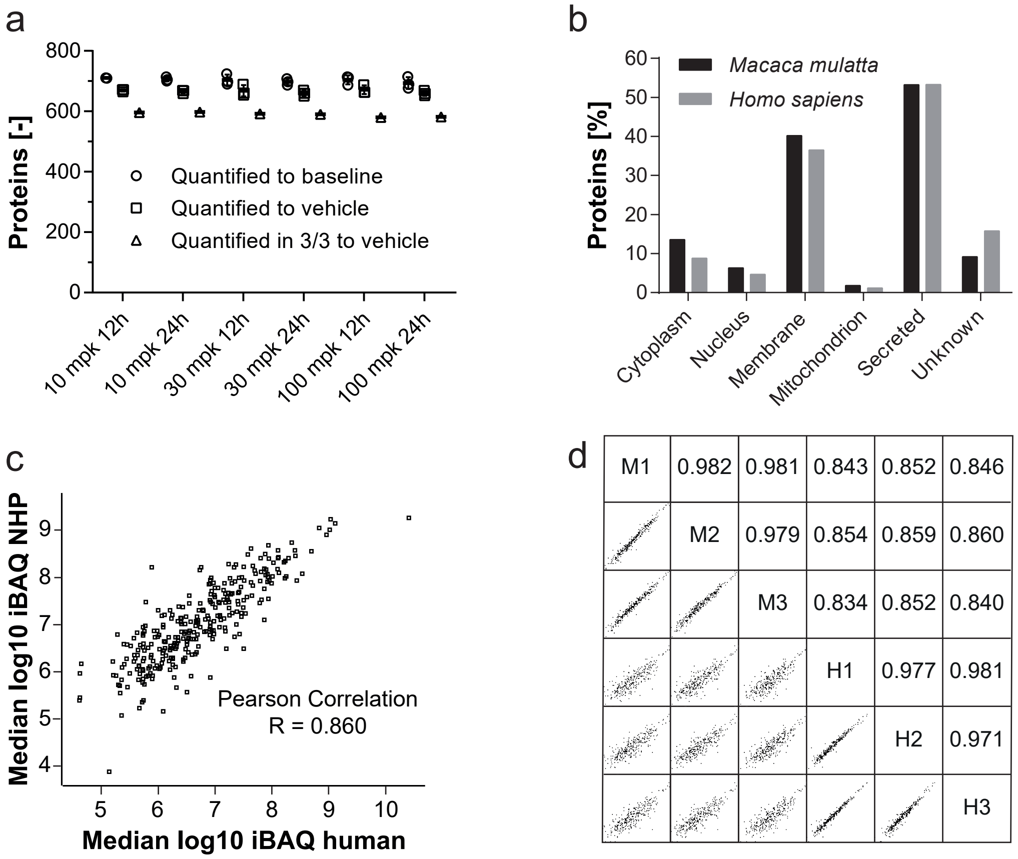

Supplementary Figure S1: CSF proteomics of MBI-4 or vehicle-treated NHP

a On average, more than 700 proteins could be quantified compared to baseline for each experiment (N=3 per condition). Around 600 proteins were detected in all three experiments and were subjected to statistical analysis for differential protein abundance. **b** The distribution of identified NHP proteins (Macaca mulatta) according to UniProt subcellular locations is very similar to previously acquired data of human CSF on the same mass spectrometric system[21]. **c** Pearson correlation of the average log10-transformed iBAQ (intensity based absolute quantification) values of NHP CSF at baseline and human reference data analyzed on the same instrument[21] indicate a good correlation. Each dot represents one protein. Only proteins with at least 3 peptides were considered for the comparison. **d** Single plots of log10-transformed iBAQ intensities of 3 NHP CSF samples at baseline (M1-M3) compared with human CSF samples (H1-H3). The Pearson correlations R are indicated in the related boxes above.
