# Supplementary material for: The Alzheimer’s disease-linked protease BACE1 modulates neuronal IL-6 signaling through shedding of the receptor gp130
Source: Mol Neurodegener. 2023 Feb 21;18:13. doi: 10.1186/s13024-023-00596-6 (PMC9942414; doi:10.1186/s13024-023-00596-6)
Supplement: Supplementary file 5 — Additional file 5: Supplementary Fig. S2. Extended plots for NHP CSF proteomics in response to MBI-4 and verubecestat. [file 13024_2023_596_MOESM5_ESM.pdf]

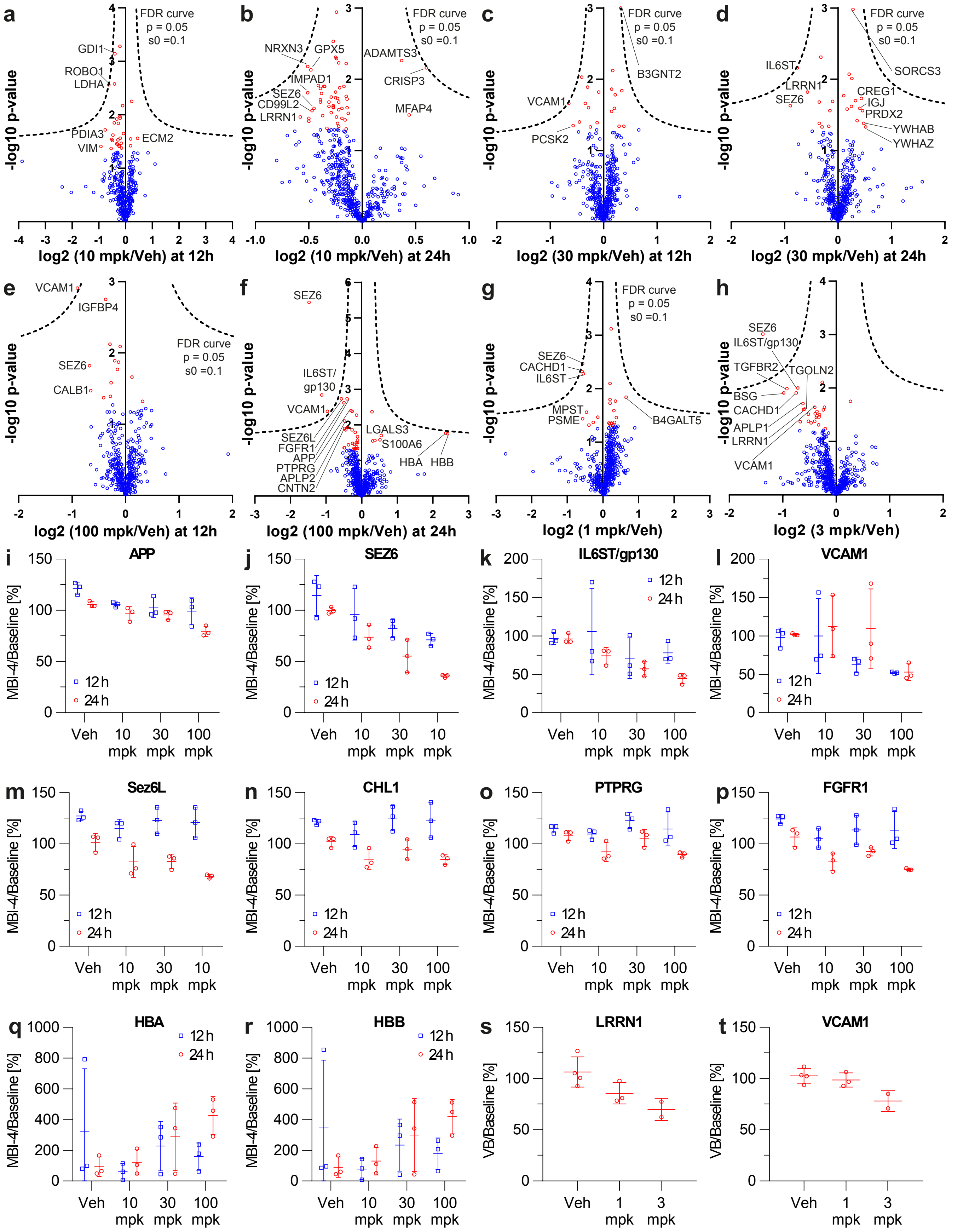

Supplementary Figure S2: Extended plots for NHP CSF proteomics in response to MBI-4 and verubecestat

a-f Volcano plots of the 3 different applied doses of the BACE inhibitor MBI-4 in comparison to the vehicle control at 12 and 24 h. The volcano plot for 100 mpk MBI-4 at 24h (**f**) is the same as illustrated in Fig. 1 to have a complete overview of all comparisons. **g-h** Volcano plots of the 2nd BACE inhibition experiment in NHPs using 1 mpk (**g**) and 3 mpk (**h**) verubecestat compared to the vehicle control are shown. Volcano plot for 3 mpk (**h**) was taken from Fig. 1 for easier comparison. **i-r** Dot plots for APP (**i**), SEZ6 (**j**), IL6ST/gp130 (**k**), VCAM1 (**l**), the validated BACE1 substrates SEZ6L (**m**) and CHL1 (**n**), as well as the substrate candidates PTPRG (**o**) and FGFR1 (**p**) at 12 and 24 h. Furthermore, dot plots of hemoglobin subunit alpha (**q**) and beta (**r**) show the tendency of an increased abundance upon BACE inhibition with MBI-4. For the 2nd BACE inhibition experiment with verubecestat, the relative abundance changes are illustrated for the validated BACE1 substrate LRRN1 (s) and VCAM1 (**t**).
