# Supplementary material for: The Alzheimer’s disease-linked protease BACE1 modulates neuronal IL-6 signaling through shedding of the receptor gp130
Source: Mol Neurodegener. 2023 Feb 21;18:13. doi: 10.1186/s13024-023-00596-6 (PMC9942414; doi:10.1186/s13024-023-00596-6)
Supplement: Supplementary file 6 — Additional file 6: Supplementary Fig. S3. Pharmacokinetics and -dynamics of NHP treated with verubecestat. [file 13024_2023_596_MOESM6_ESM.pdf]

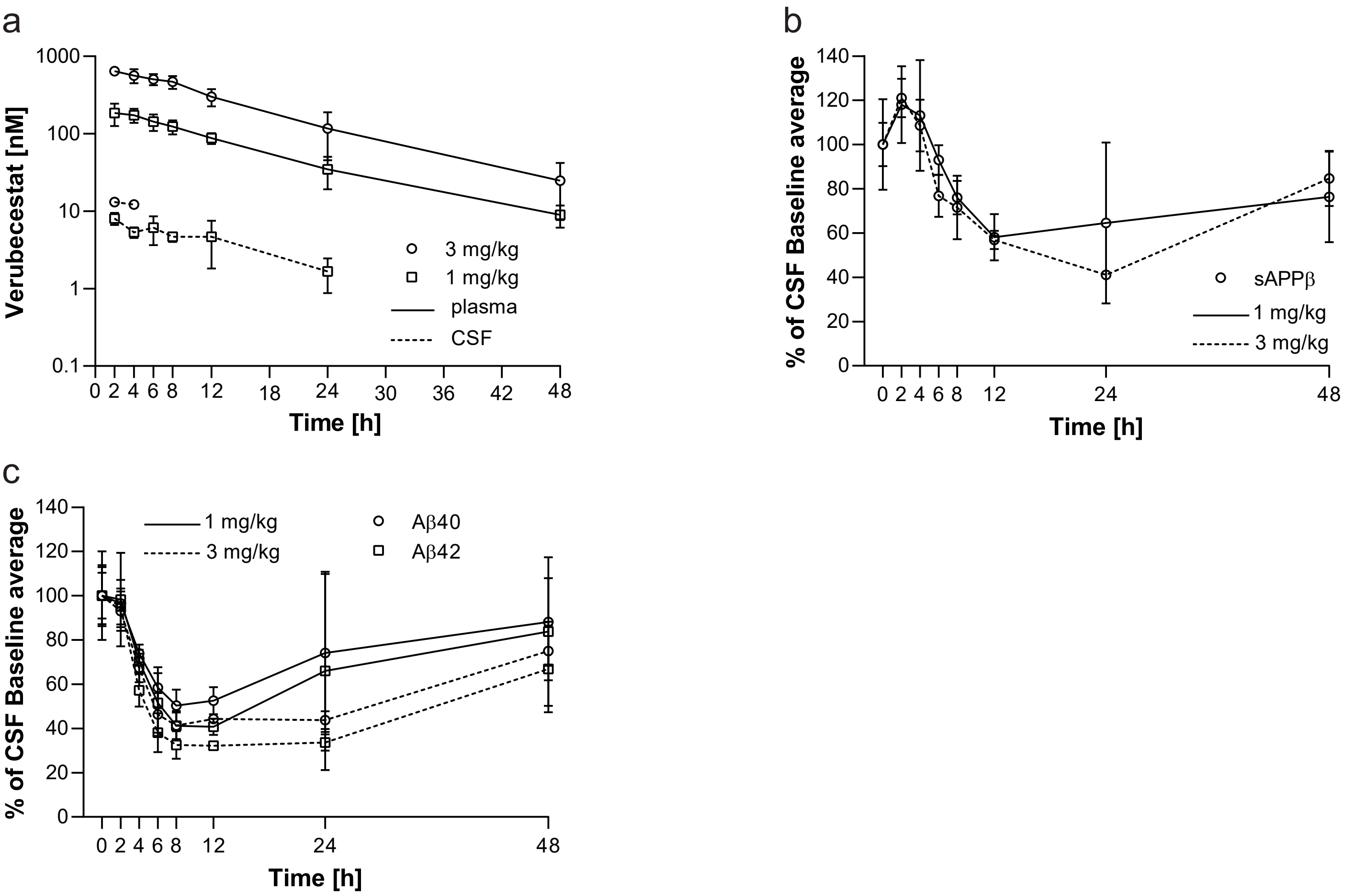

Supplementary Figure S3: Pharmacokinetics and -dynamics of NHP treated with verubecestat

Cisterna magna ported Rhesus monkeys were orally dosed with verubecestat at 1 or 3 mg/kg. **a** Total concentration of verubecestat in plasma and CSF. CSF concentrations were below the limit of quantitation of the assay (10 nM) for the majority of 3 mg/kg CSF samples. The LOQ for 1 mg/kg CSF samples was 1 nM. **b** CSF sAPPβ profiles following single oral doses of verubecestat at 1 or 3 mg/kg. **c** Baseline-normalized CSF Aβ40 and Aβ42 profiles following a single oral dose of verubecestat at 1 or 3 mg/kg. Shown are mean ± SD from N=3 biological replicates.
