# Supplementary material for: The Alzheimer’s disease-linked protease BACE1 modulates neuronal IL-6 signaling through shedding of the receptor gp130
Source: Mol Neurodegener. 2023 Feb 21;18:13. doi: 10.1186/s13024-023-00596-6 (PMC9942414; doi:10.1186/s13024-023-00596-6)
Supplement: Supplementary file 7 — Additional file 7: Supplementary Fig. S4. Full Western blots of sSEZ6 in human CSF after BACE inhibition. [file 13024_2023_596_MOESM7_ESM.pdf]

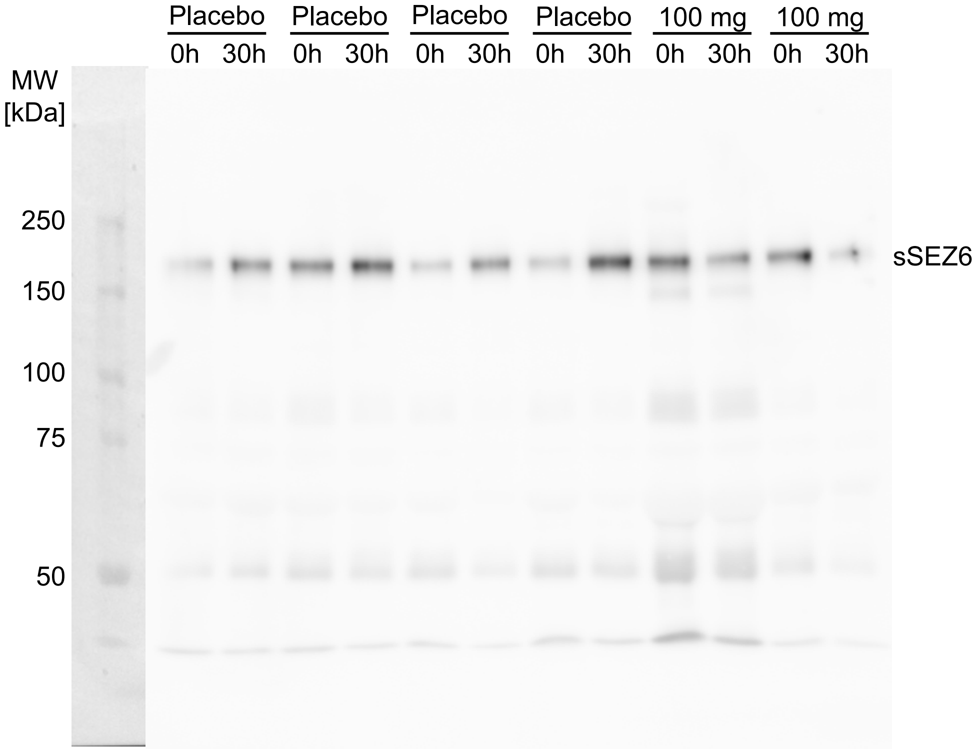

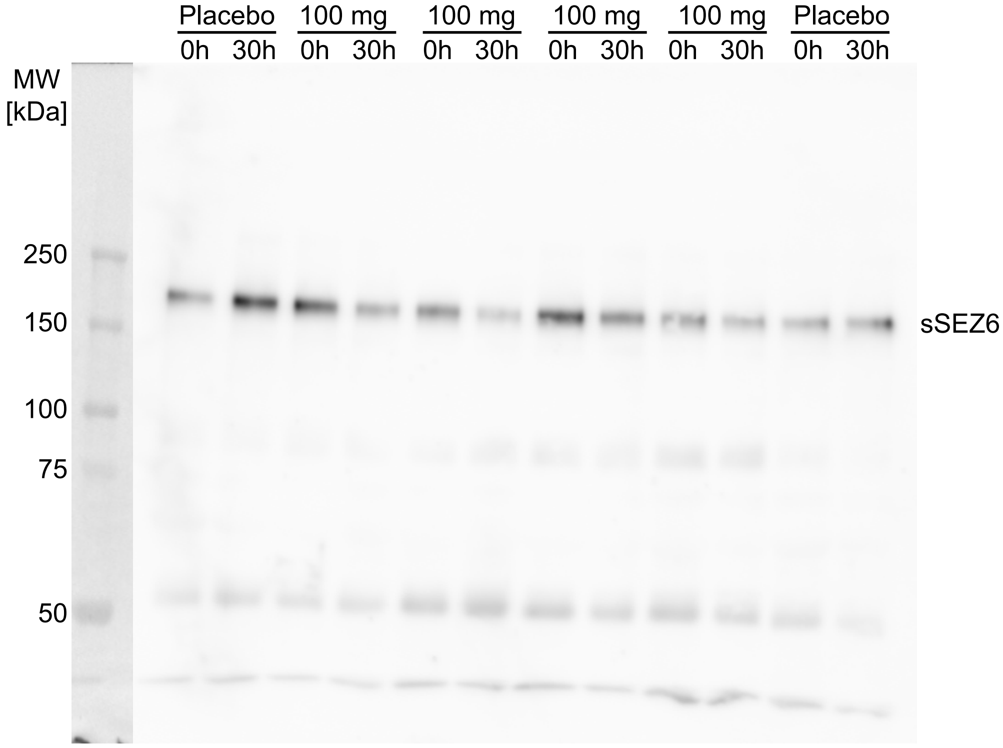

Supplementary Figure S4: Full Western blots of sSEZ6 in human CSF after BACE inhibition.

Western Blots of CSF sSEZ6 from participants of a clinical phase 1 study treated with placebo or 100 mg verubecestat (N=6 per condition). A volume of 15 µL of CSF was loaded per lane. The baseline samples (0 h) and post-dose samples at 30 h are plotted next to each other. The blots are identical to the ones shown in Fig. 2a, but show the whole membranes.
