# Supplementary material for: The Alzheimer’s disease-linked protease BACE1 modulates neuronal IL-6 signaling through shedding of the receptor gp130
Source: Mol Neurodegener. 2023 Feb 21;18:13. doi: 10.1186/s13024-023-00596-6 (PMC9942414; doi:10.1186/s13024-023-00596-6)
Supplement: Supplementary file 8 — Additional file 8: Supplementary Fig. S5. Increased survival of neurons can be blocked by sgp130. [file 13024_2023_596_MOESM8_ESM.pdf]

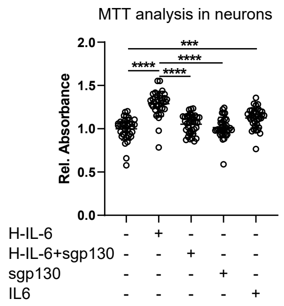

Supplementary Figure S5: Increased survival neurons can be blocked by sgp130.

MTT measurement of primary neurons at DIV12 after culture in the absence of serum-like B27 upon activation with H-IL-6, H-IL-6/sgp130, sgp130, and IL6. Shown are mean ± SD from N=36 biological replicates. One-way ANOVA with post hoc Tukey's multiple comparison test. ***: $p < 0.001$; ****: $p < 0.0001$.
